# Supplementary figures and images for: Satellite tracking of juvenile whale sharks in the Sulu and Bohol Seas, Philippines
Source: PeerJ. 2018 Jul 24;6:e5231. doi: 10.7717/peerj.5231 (PMC6063259; doi:10.7717/peerj.5231)

P - 430

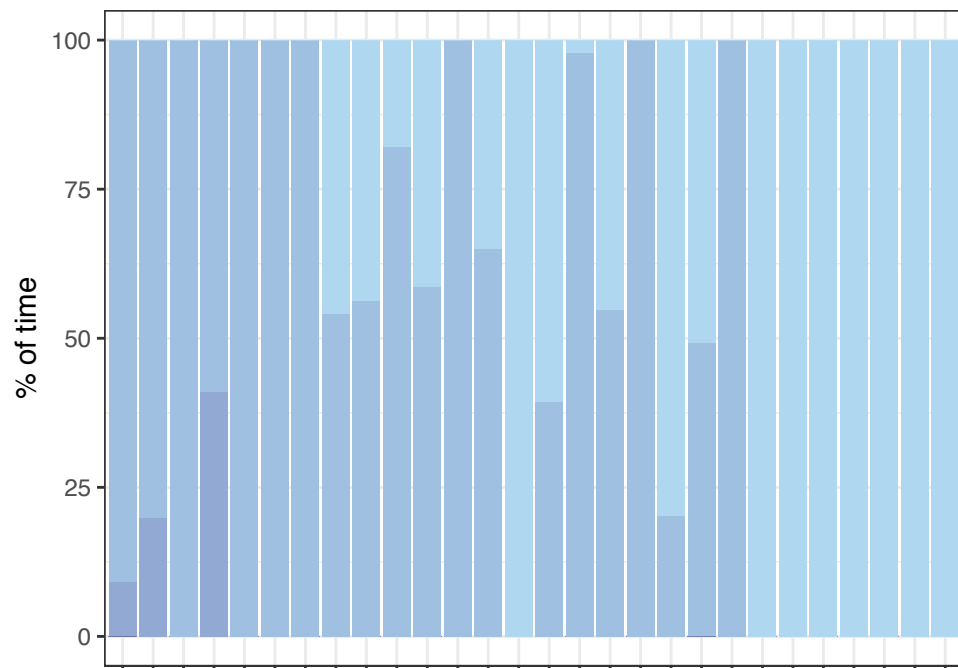

P - 430

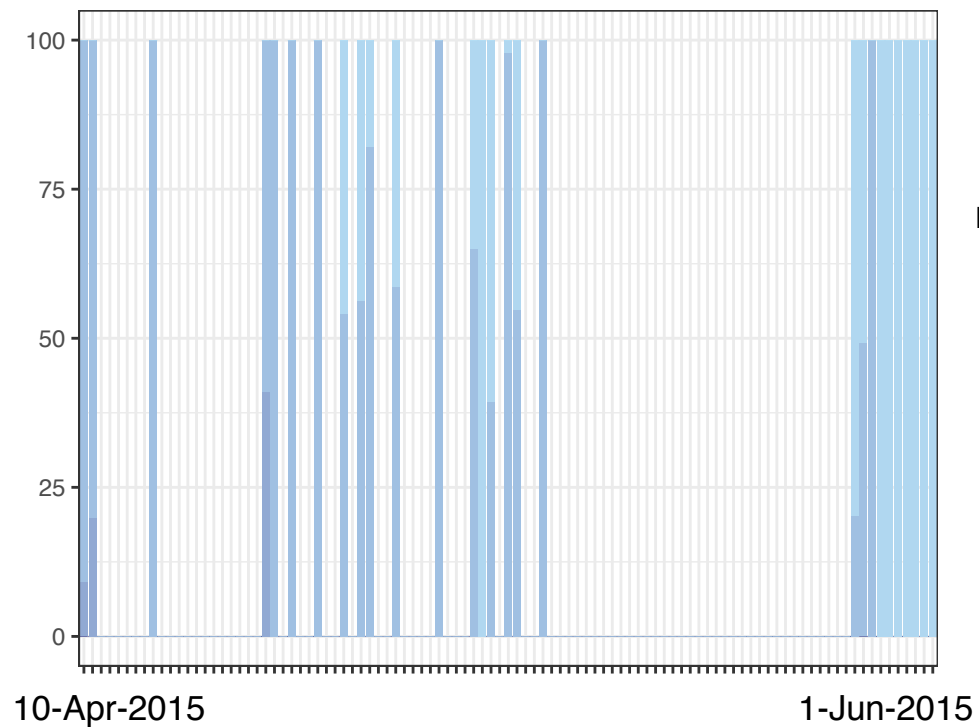

P - 430 Day

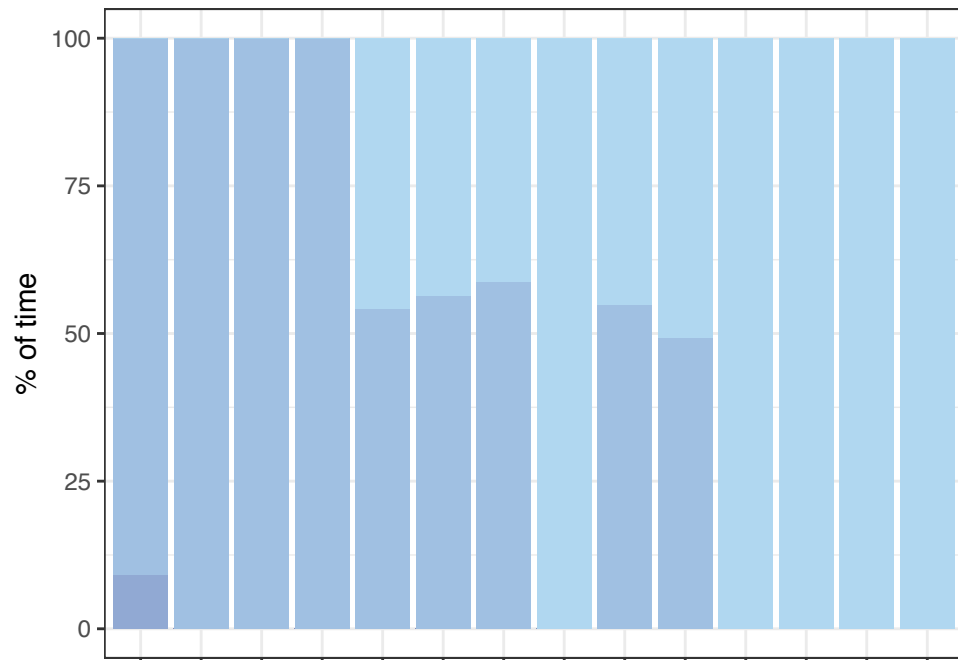

P - 430 Night

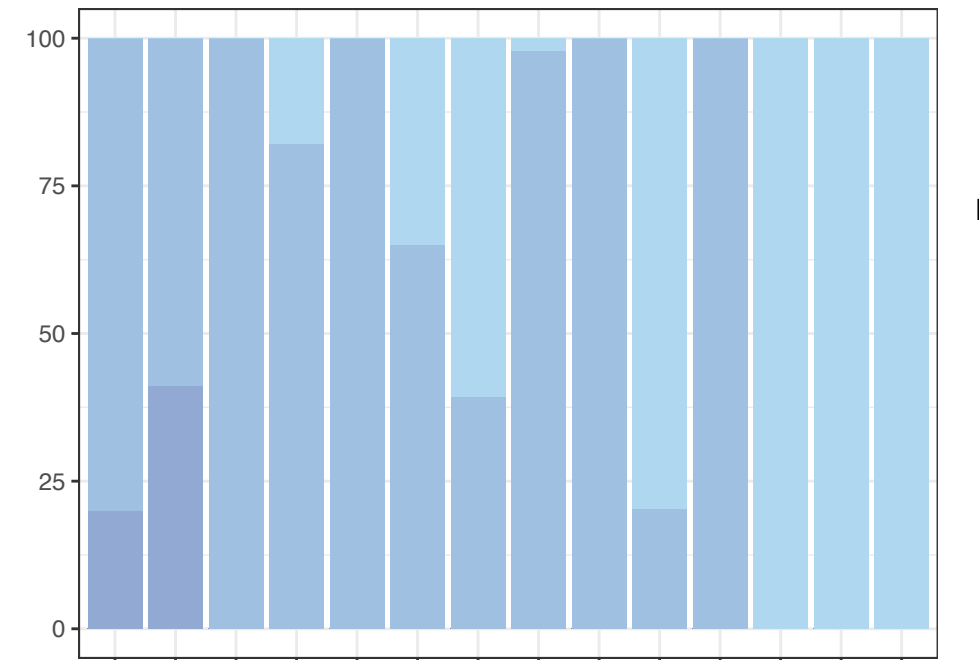

Supplement: Supplemental Information 1 [file peerj-06-5231-s001.pdf]

P - 532

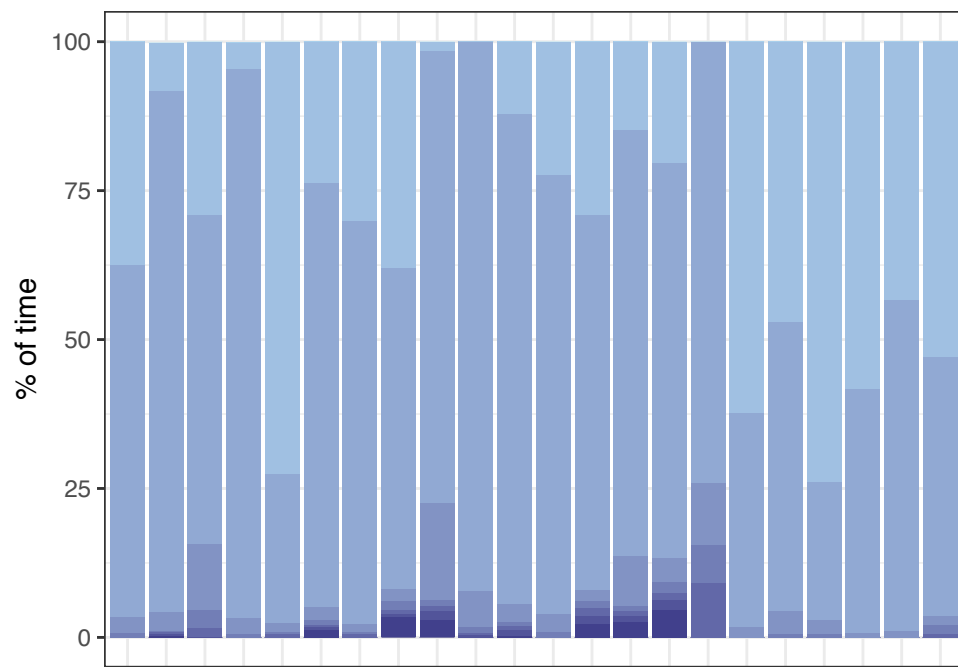

P - 532

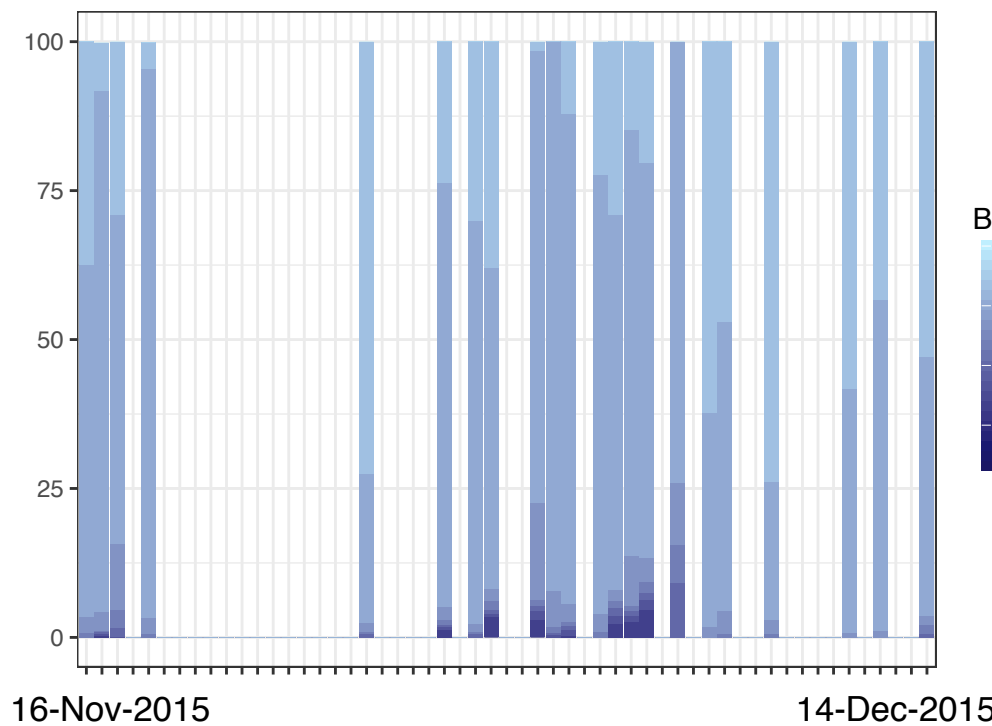

P - 532 Day

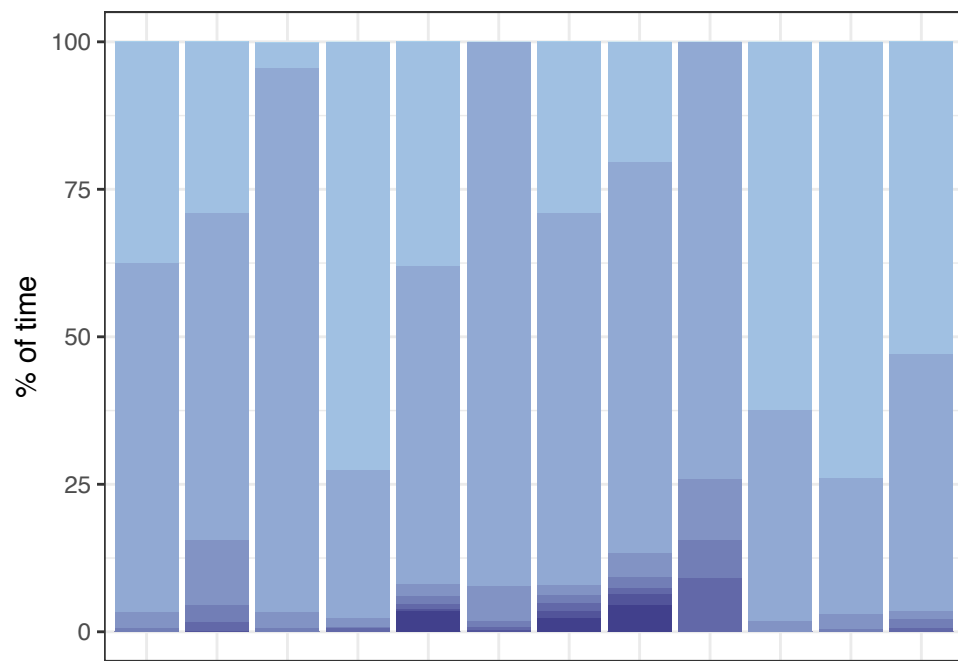

P - 532 Night

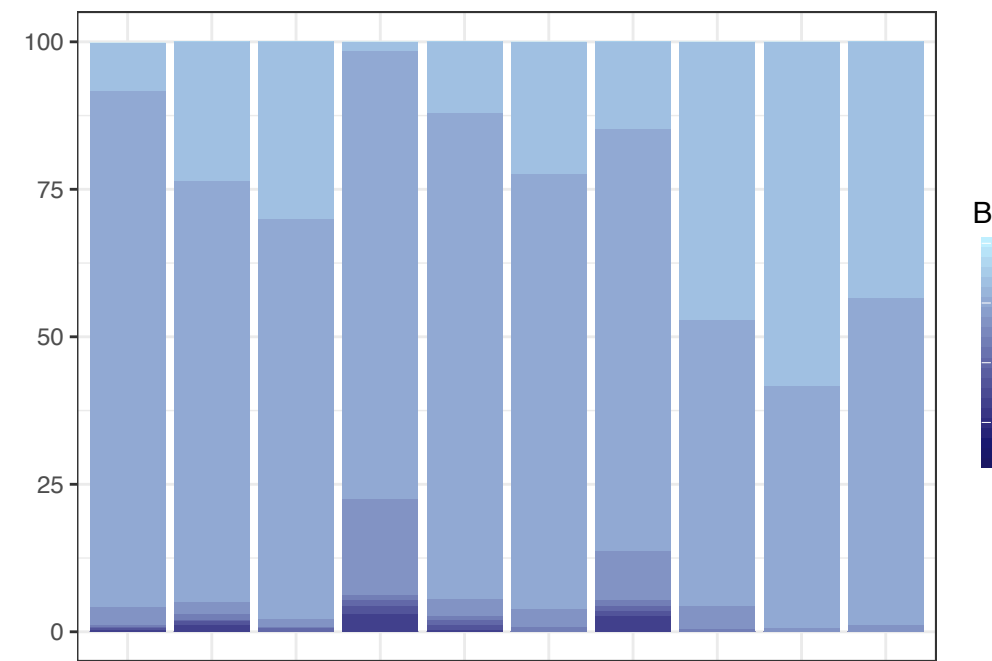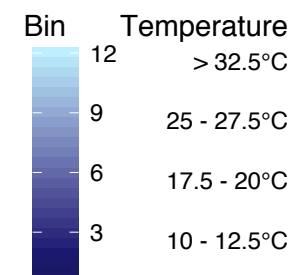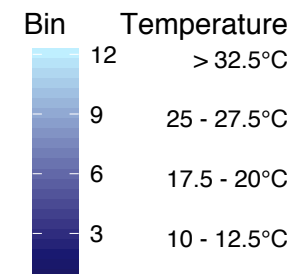

Supplement: Supplemental Information 2 [file peerj-06-5231-s002.pdf]

P - 791

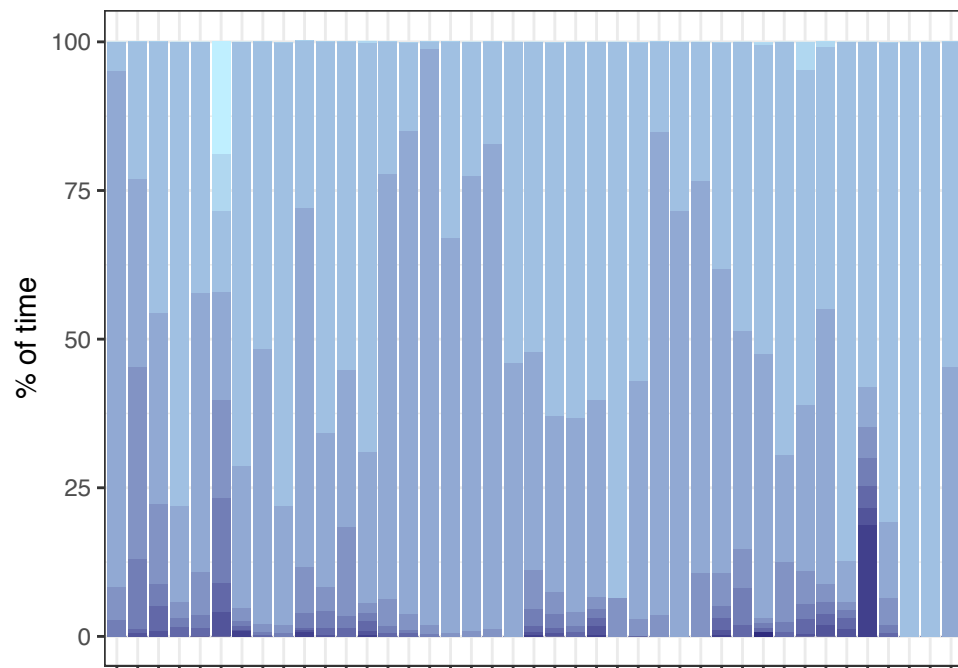

P - 791

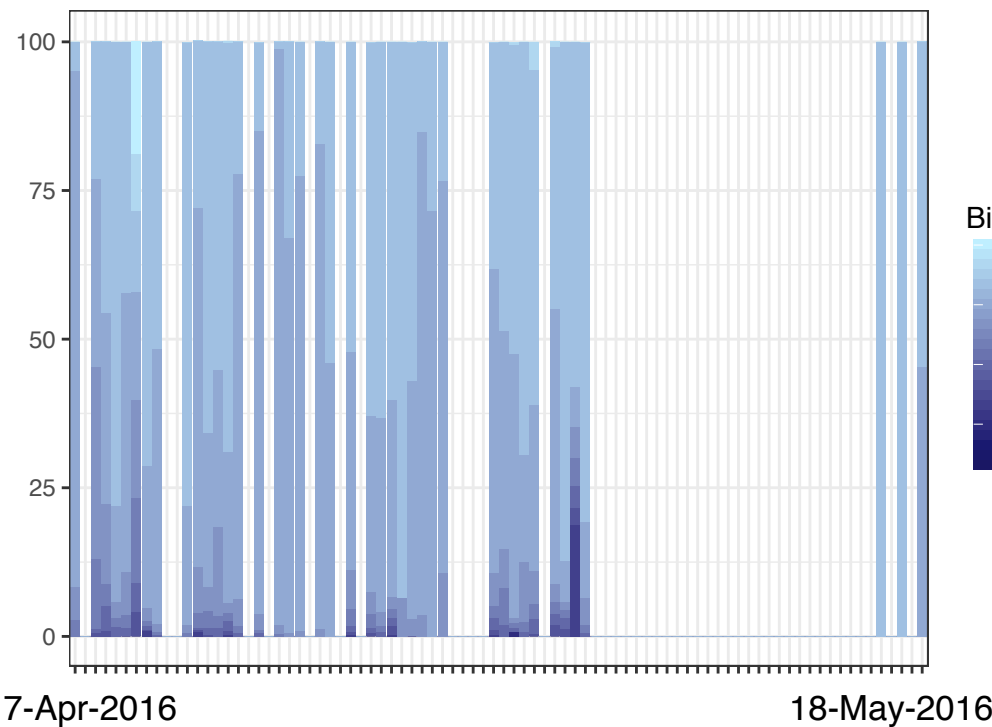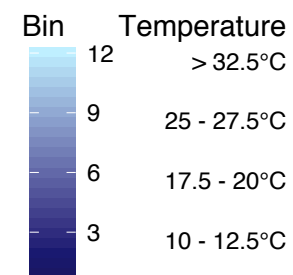

P - 791 Day

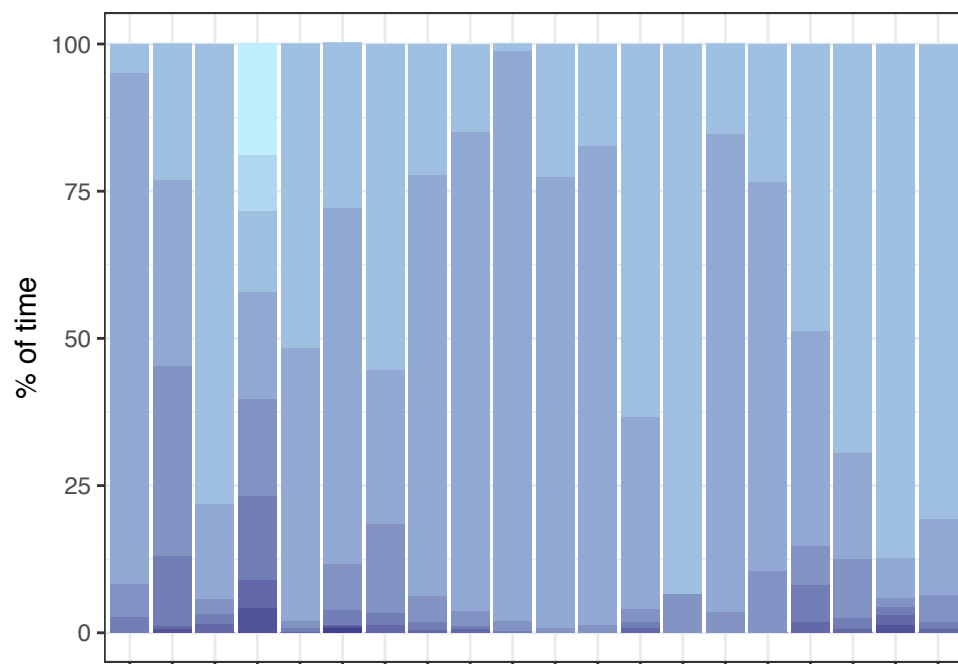

P - 791 Night

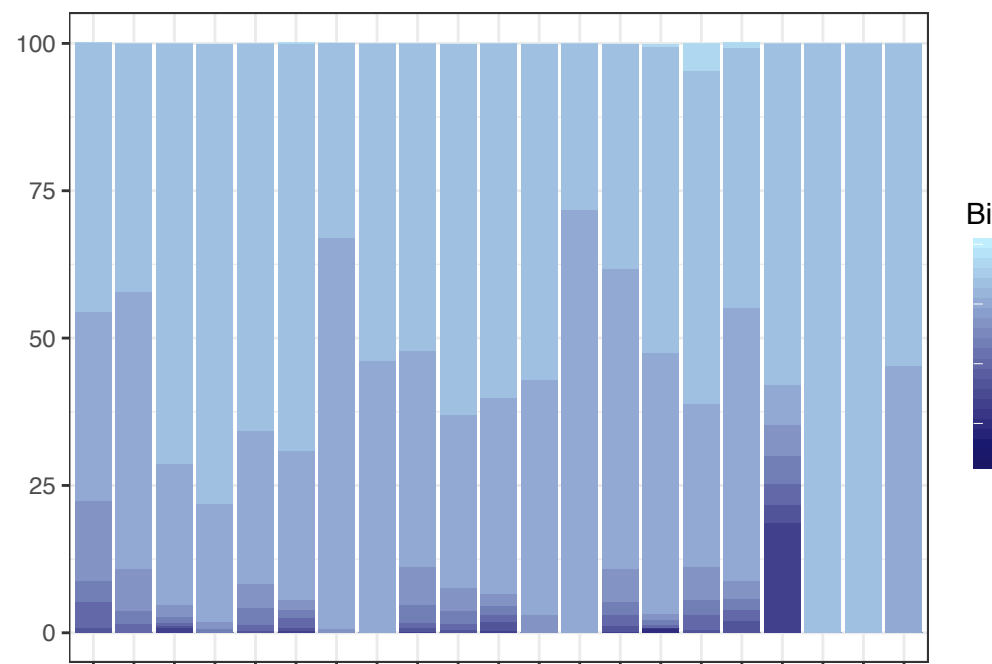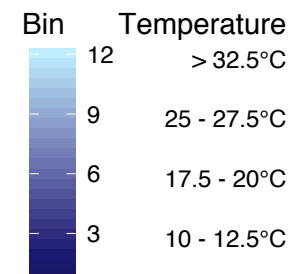

Supplement: Supplemental Information 3 [file peerj-06-5231-s003.pdf]

P – 814

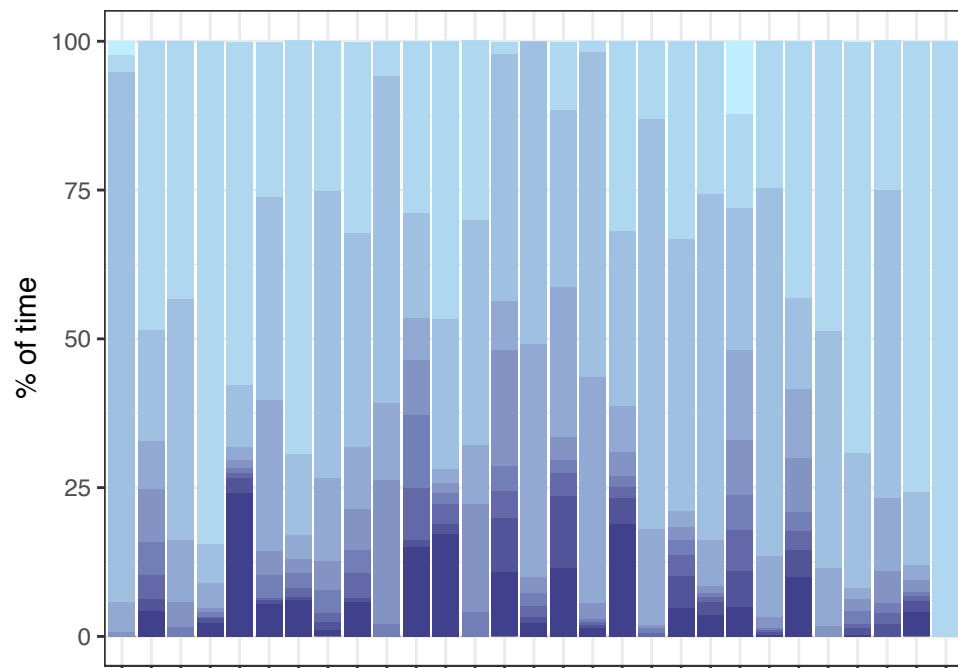

P – 814

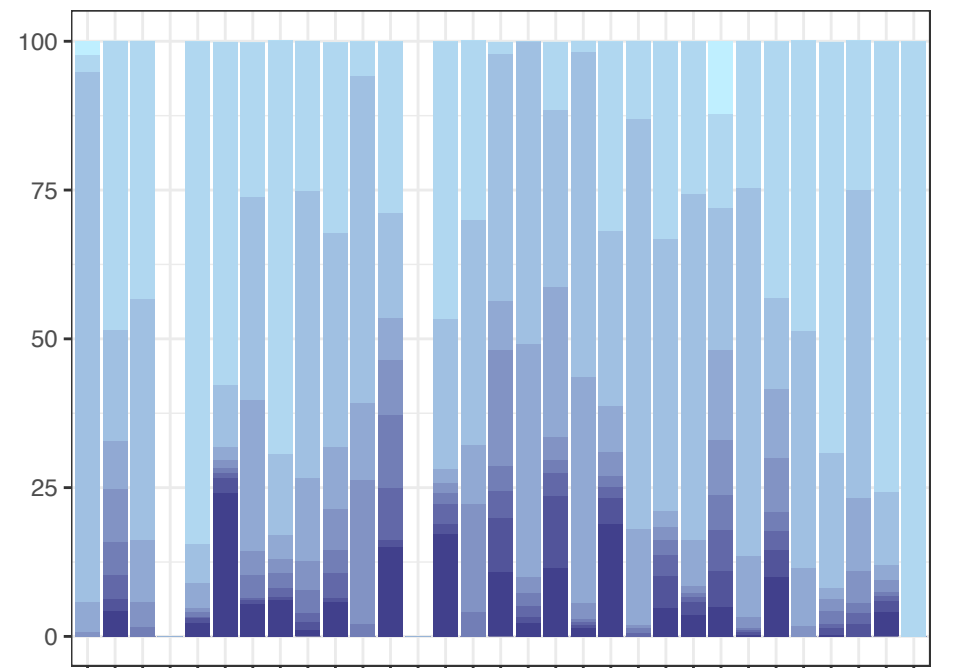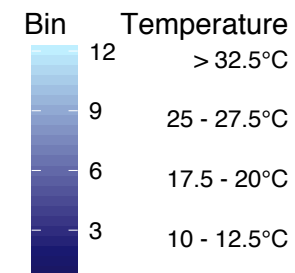

17-May-2015

1-Jun-2015

P – 814 Day

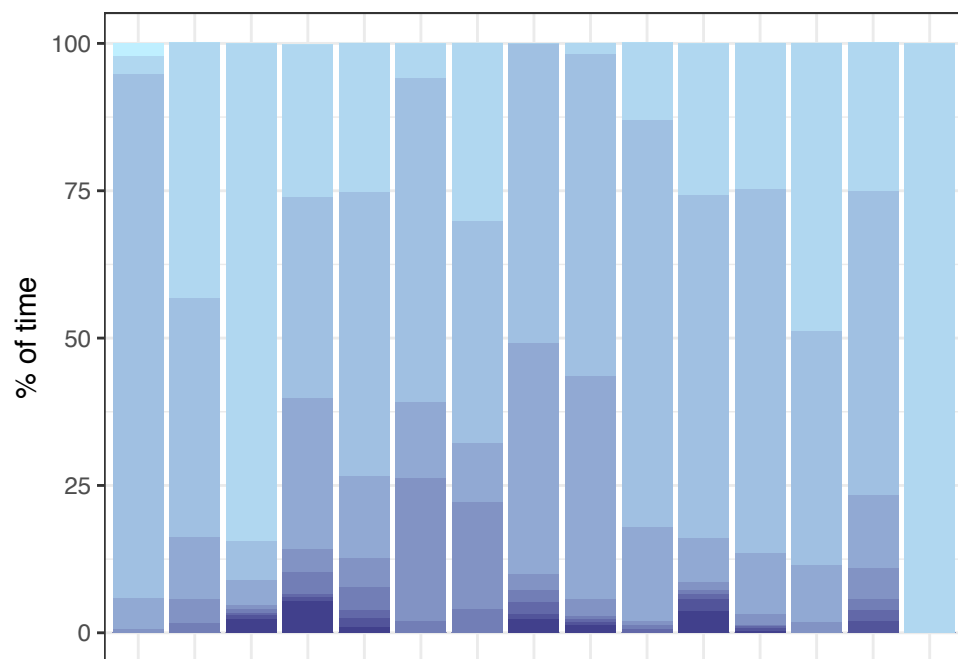

P – 814 Night

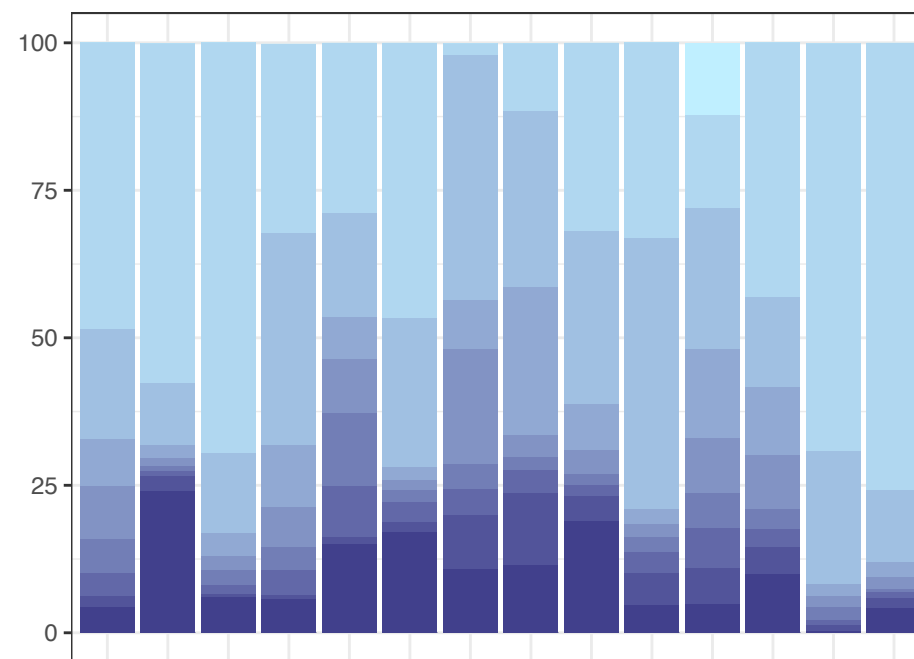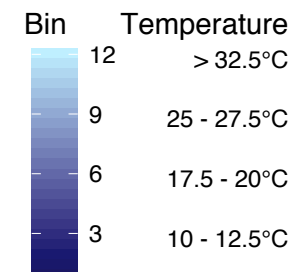

Supplement: Supplemental Information 4 [file peerj-06-5231-s004.pdf]

P - 816

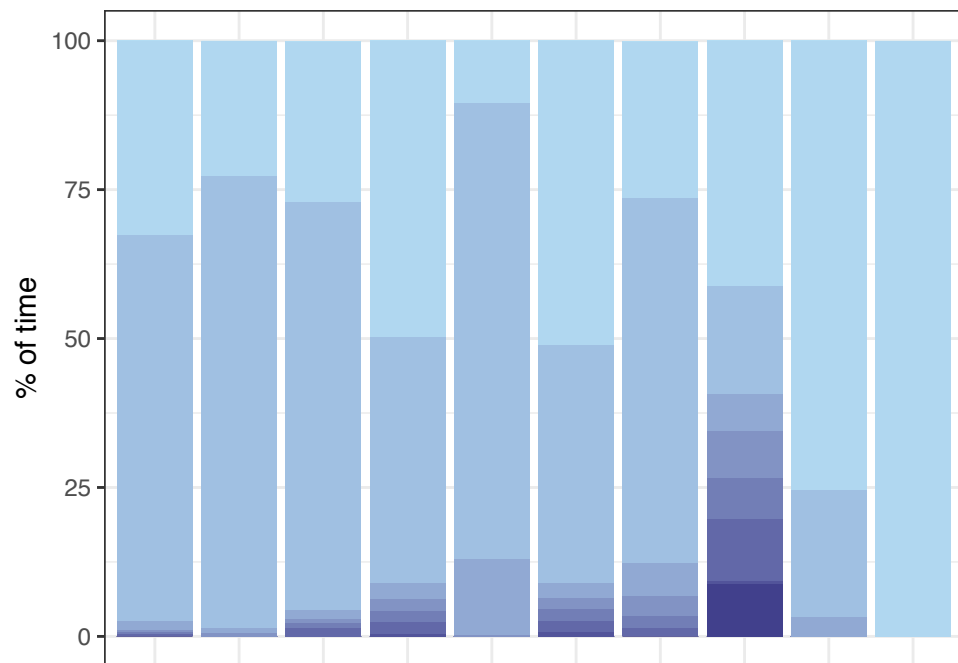

P - 816

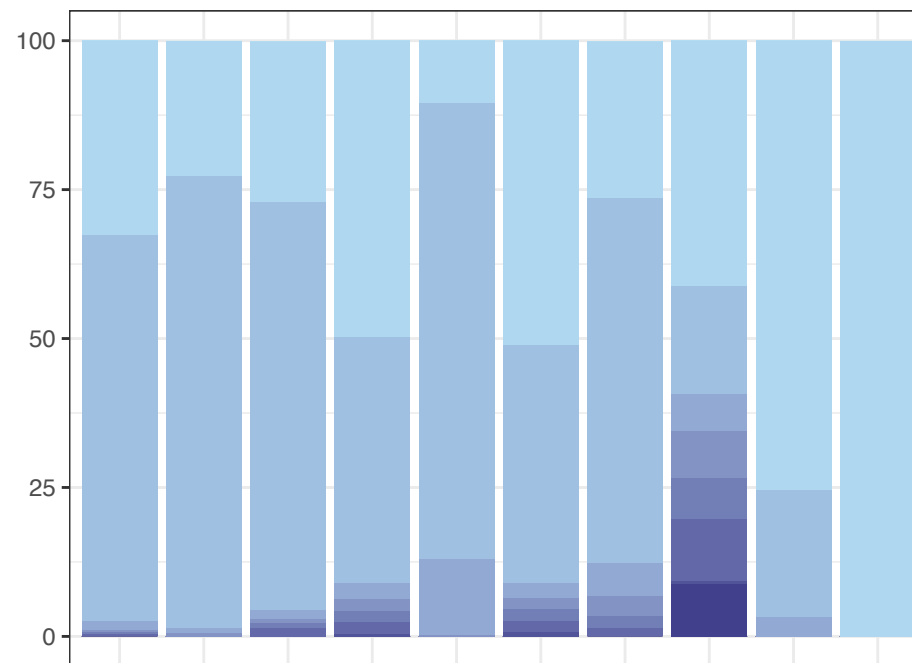

20-May-2015

25-May-2015

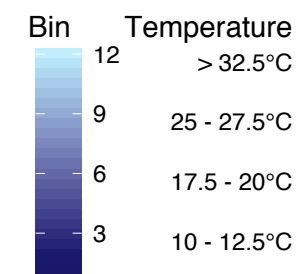

P - 816 Day

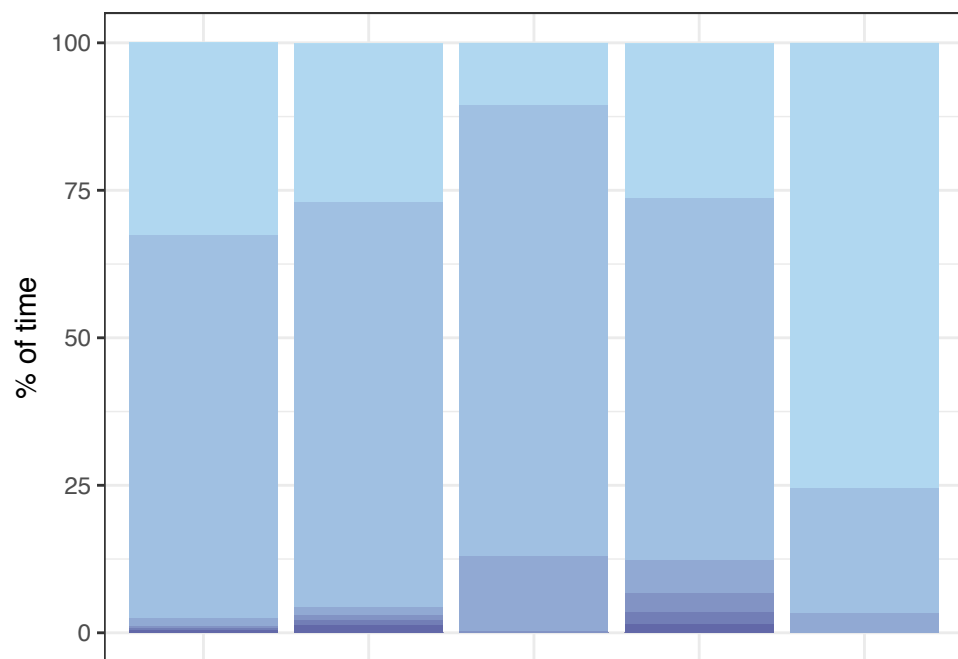

P - 816 Night

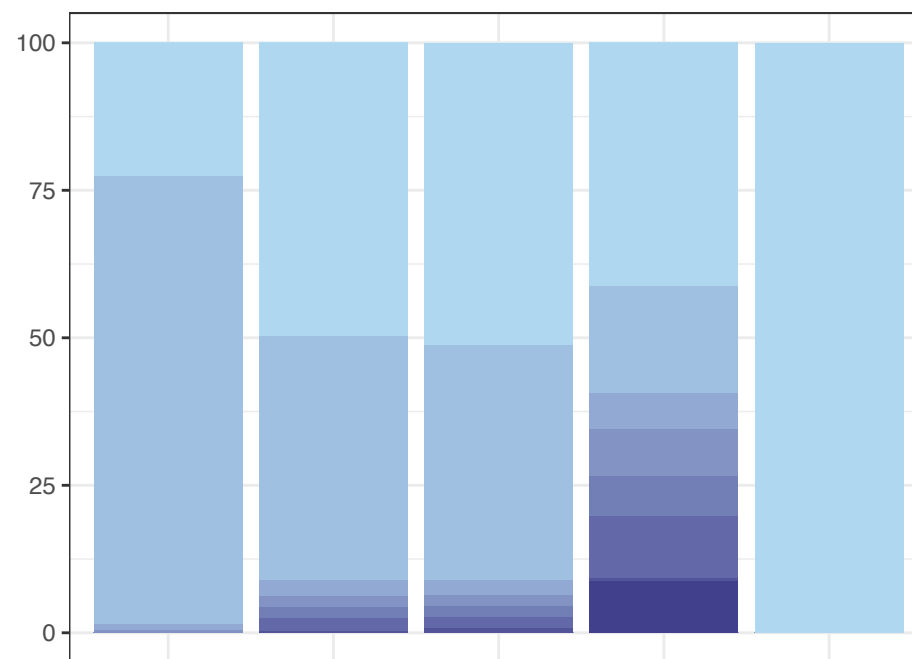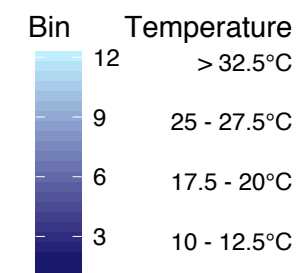

Supplement: Supplemental Information 5 [file peerj-06-5231-s005.pdf]

P - 904

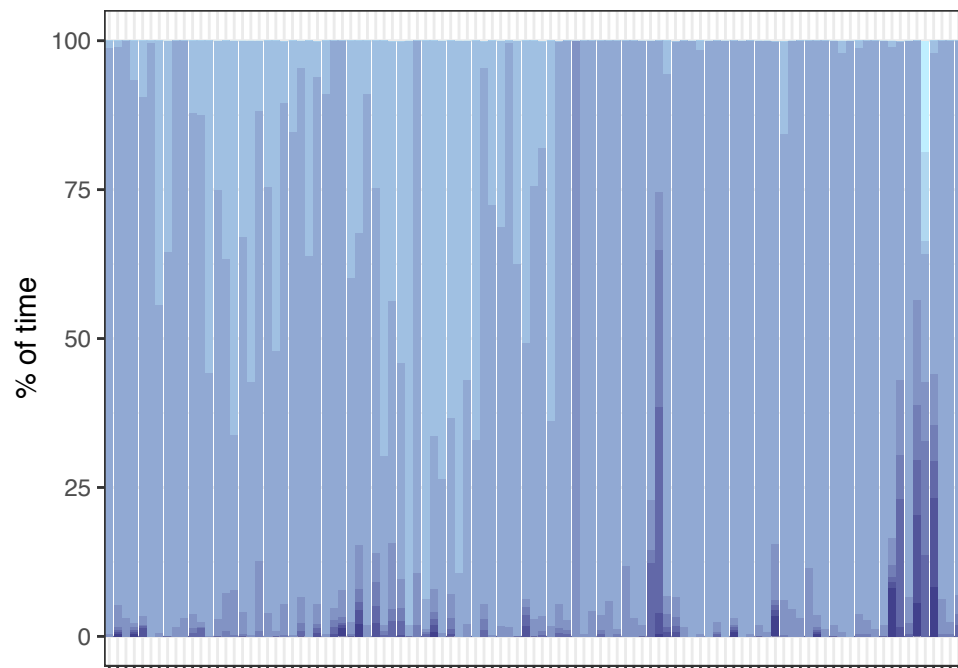

P - 904

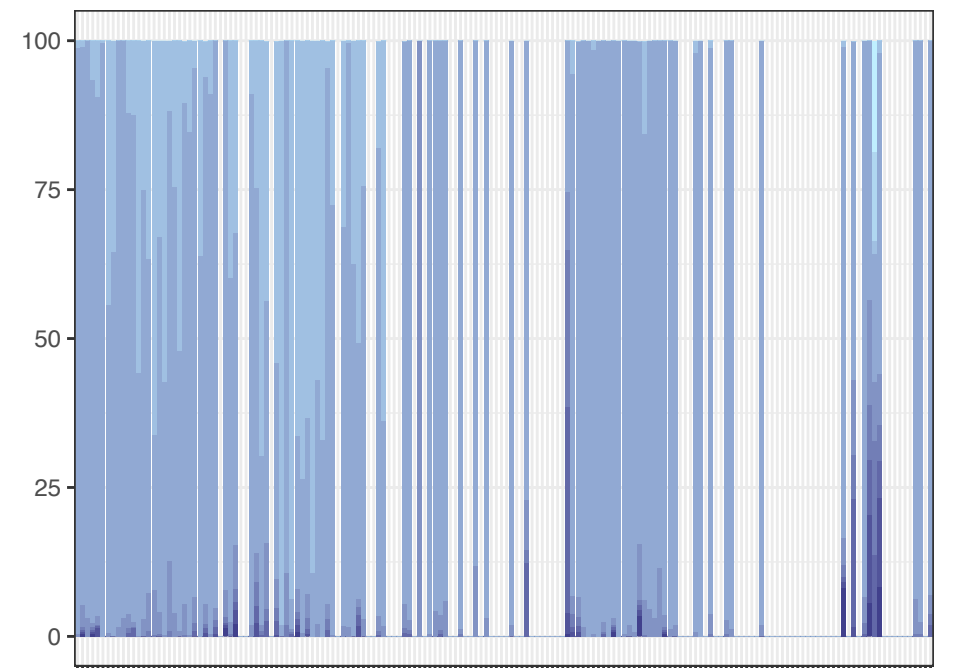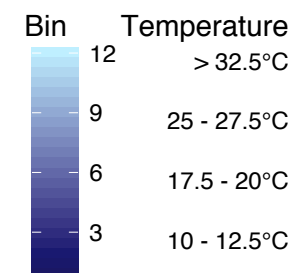

17-Nov-2015

3-Mar-2016

P - 904 Day

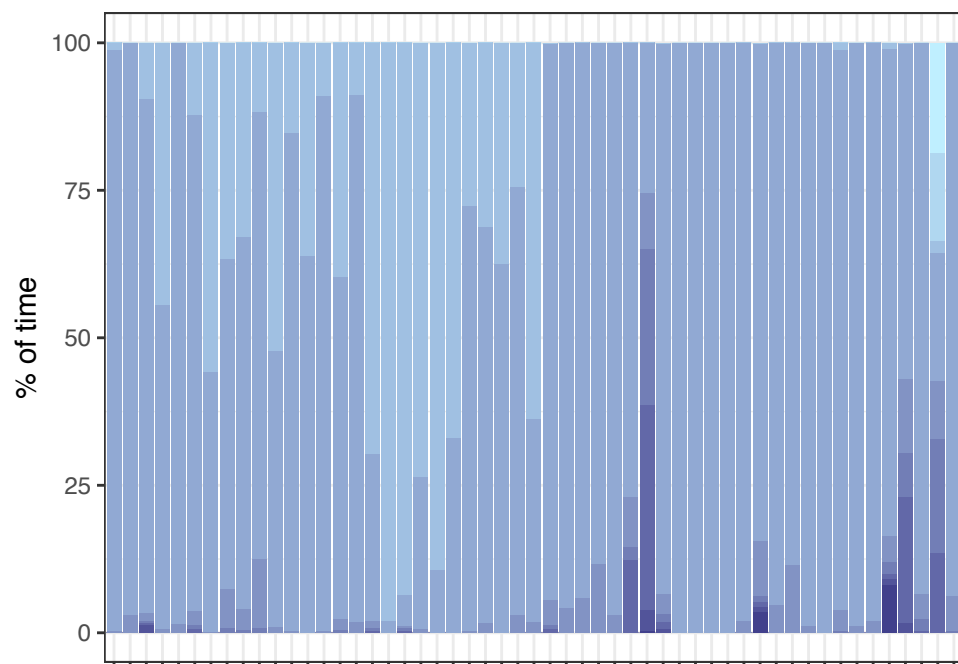

P - 904 Night

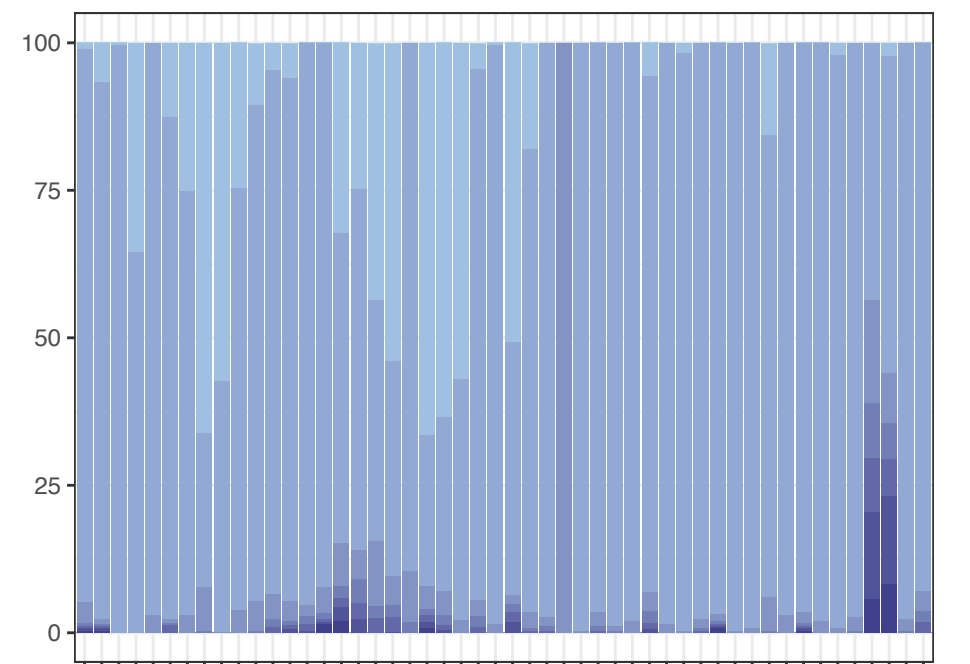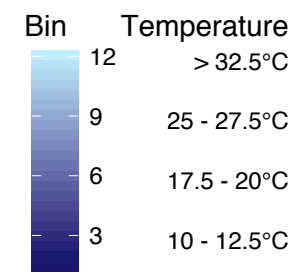

Supplement: Supplemental Information 6 [file peerj-06-5231-s006.pdf]

P - 493

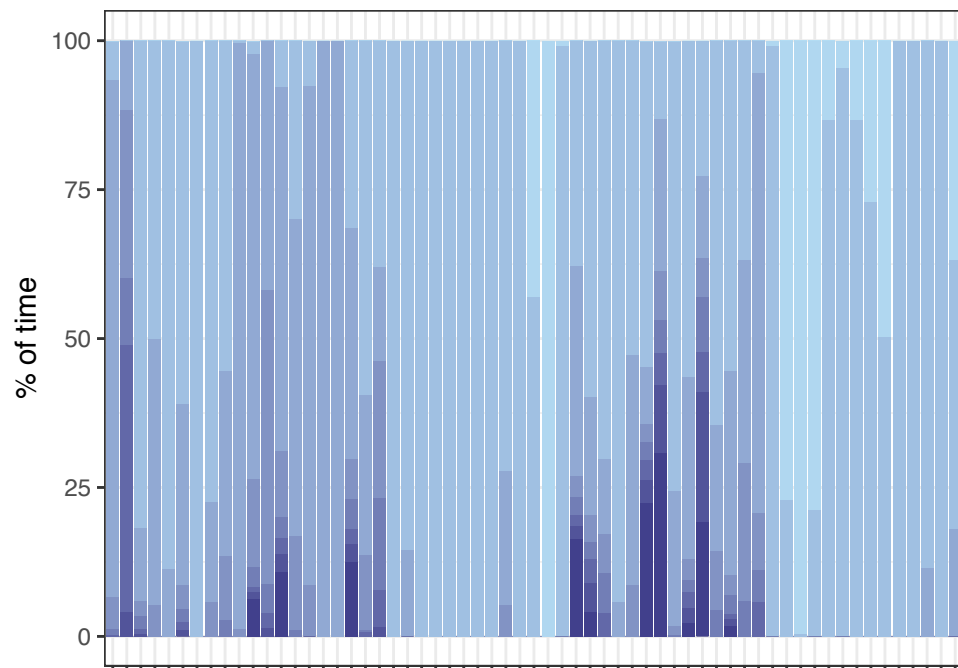

P - 493

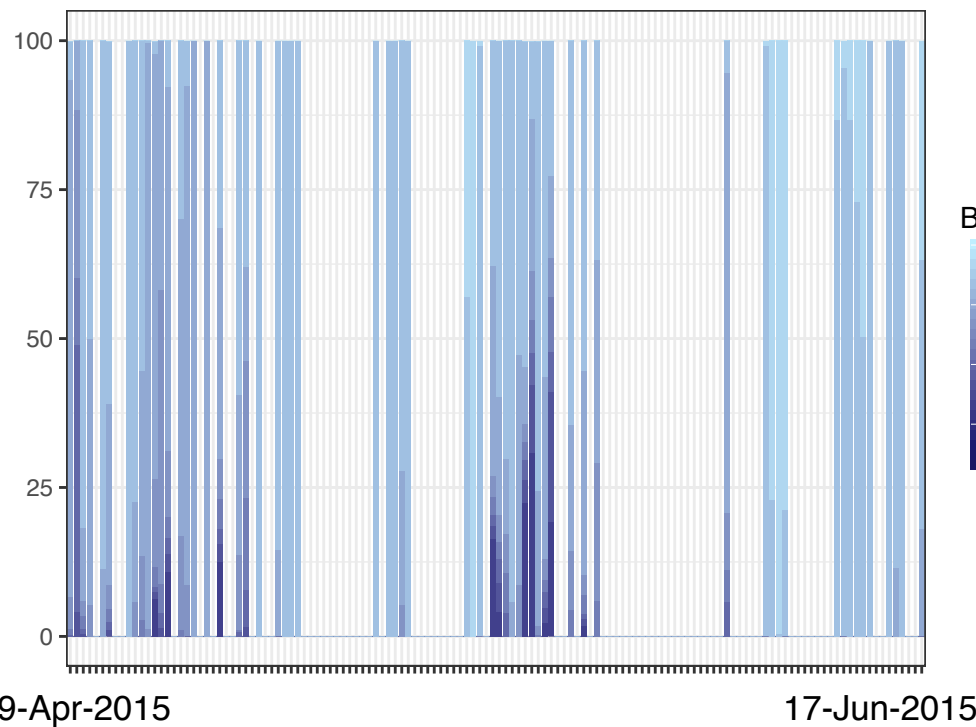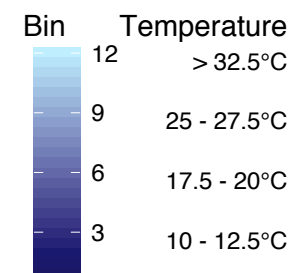

P - 493 Day

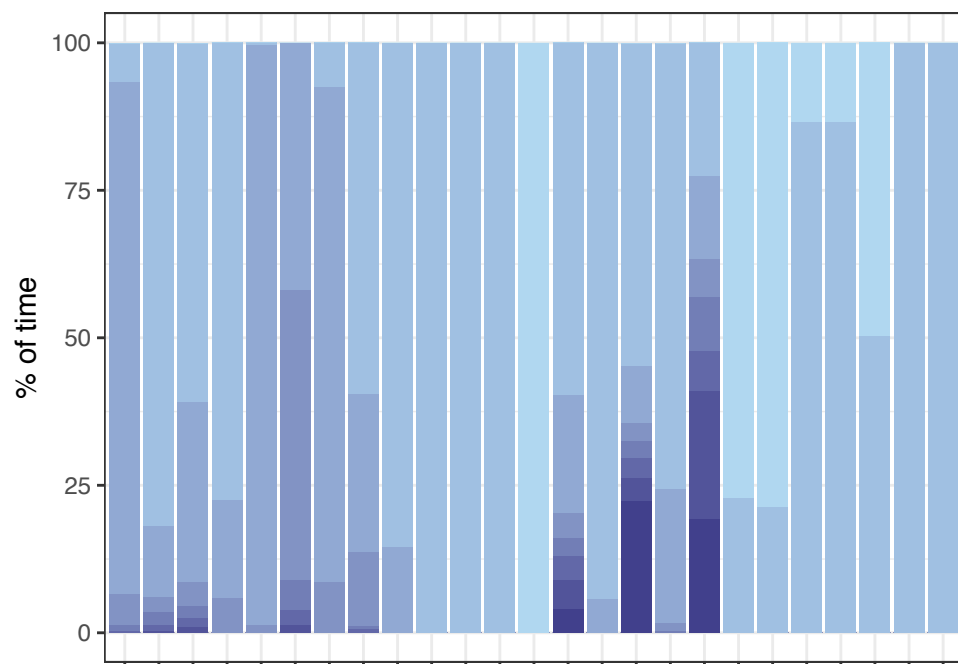

P - 493 Night

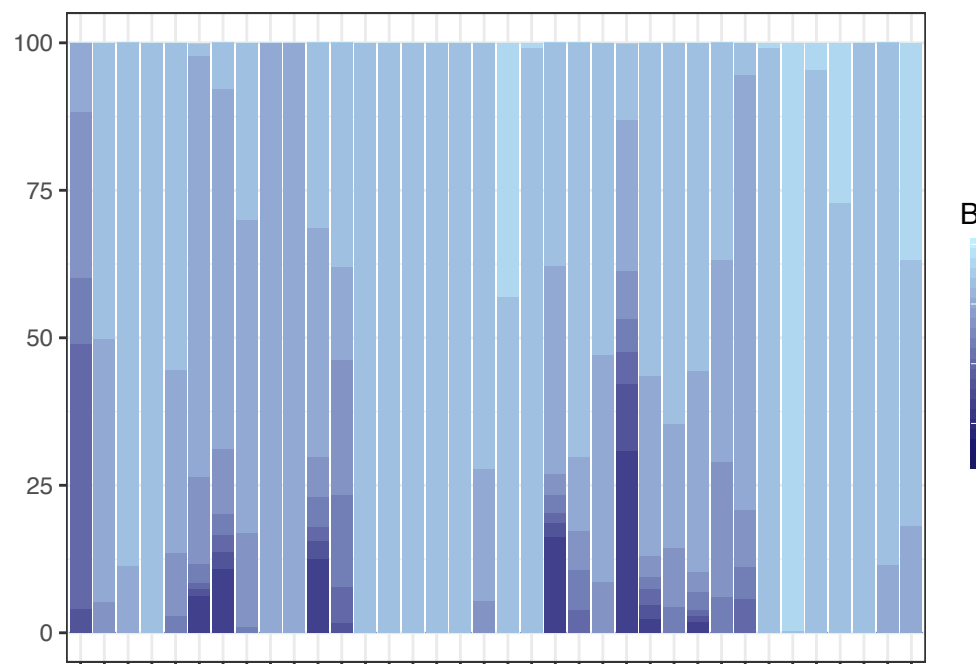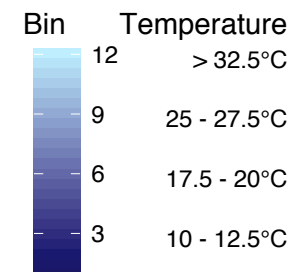

Supplement: Supplemental Information 7 [file peerj-06-5231-s007.pdf]

P - 909

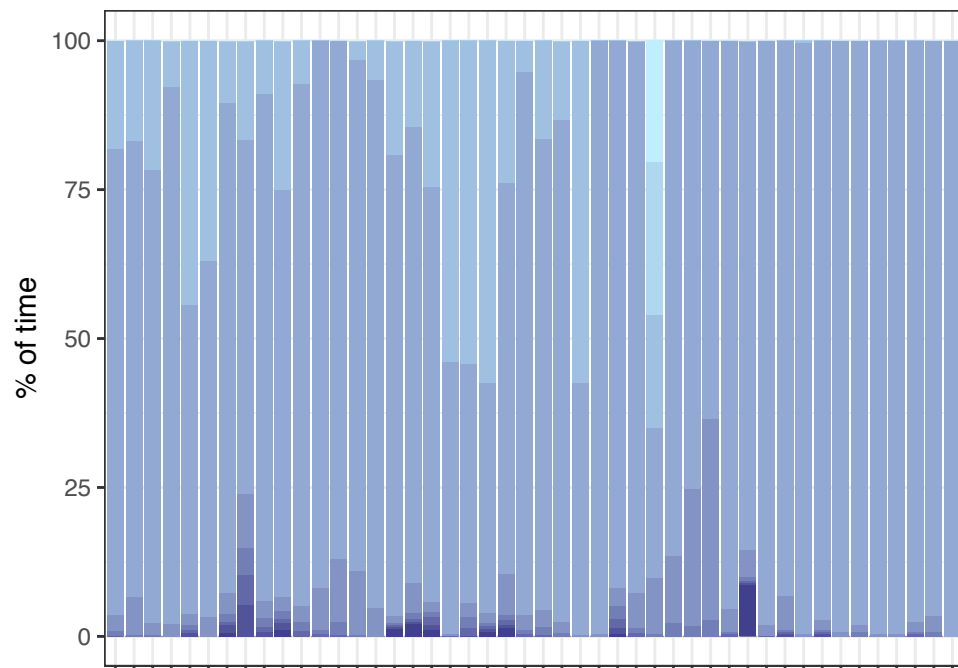

P - 909

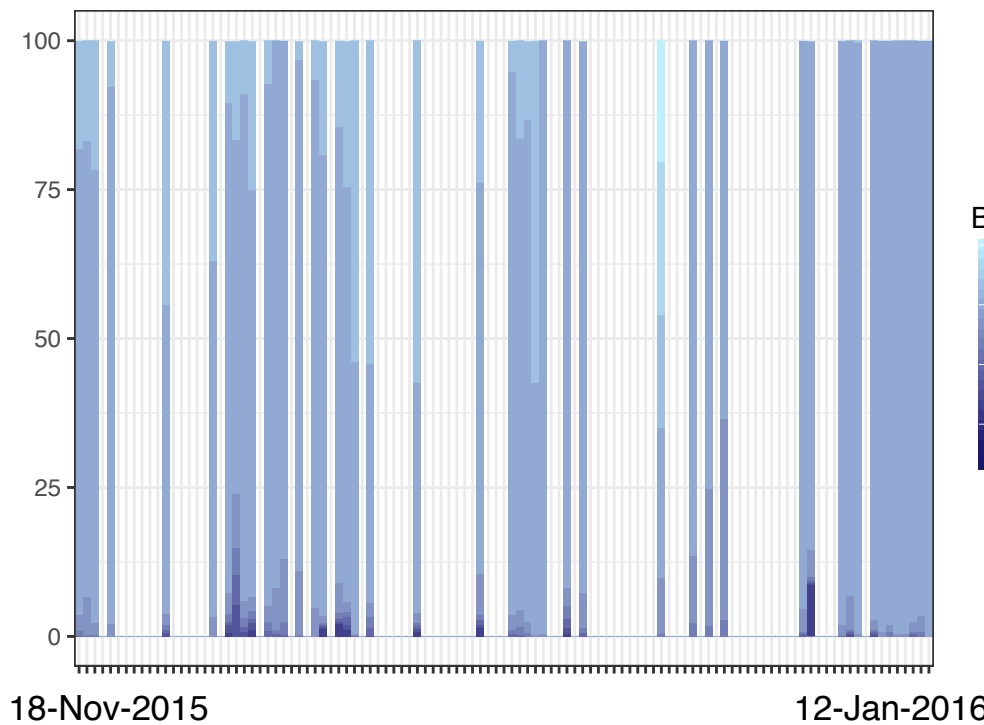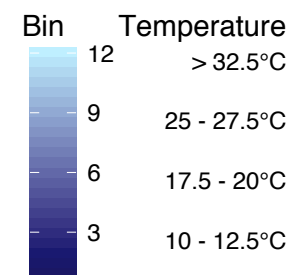

P - 909 Day

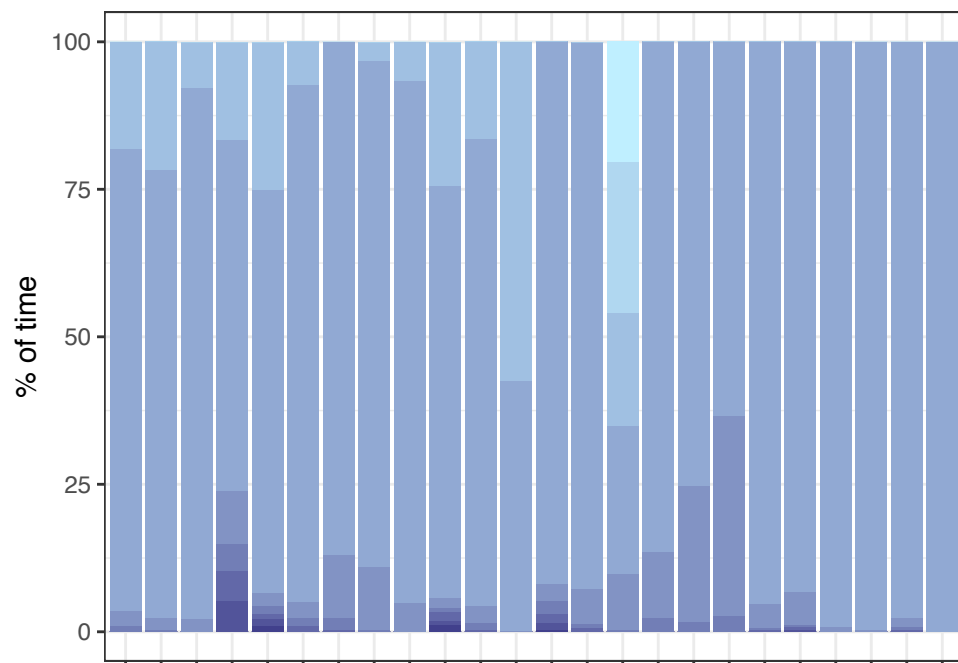

P - 909 Night

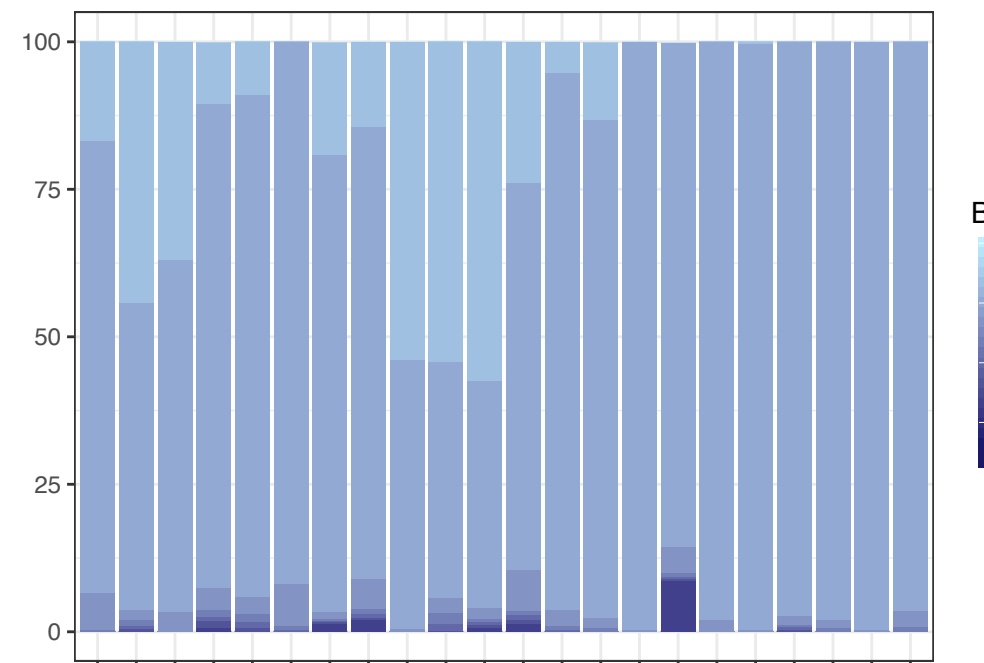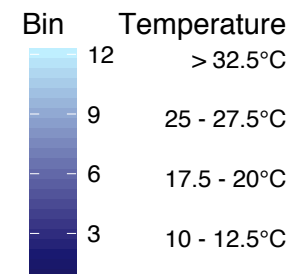

Supplement: Supplemental Information 8 [file peerj-06-5231-s008.pdf]

P - 821

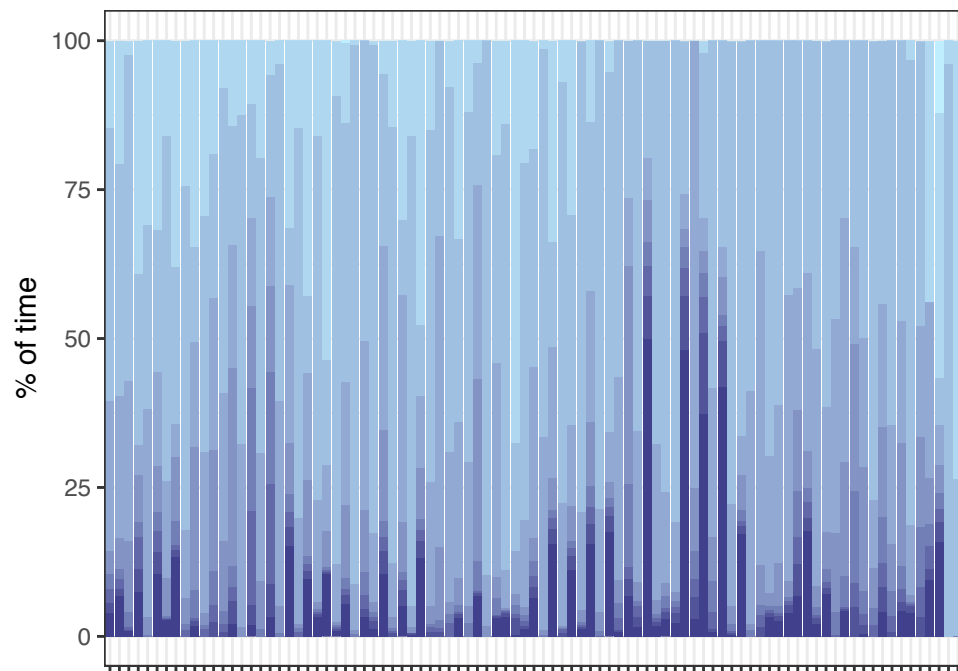

P - 821

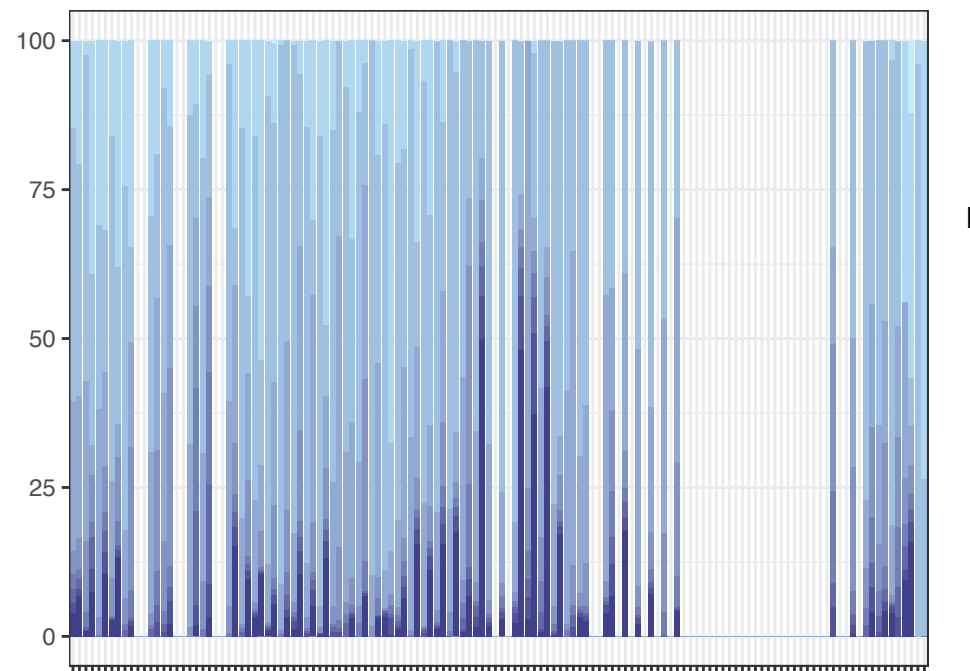

23-May-2015

28-Jul-2015

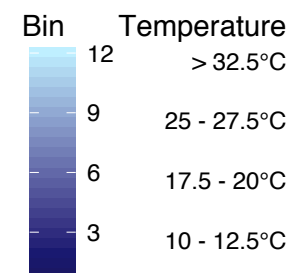

P - 821 Day

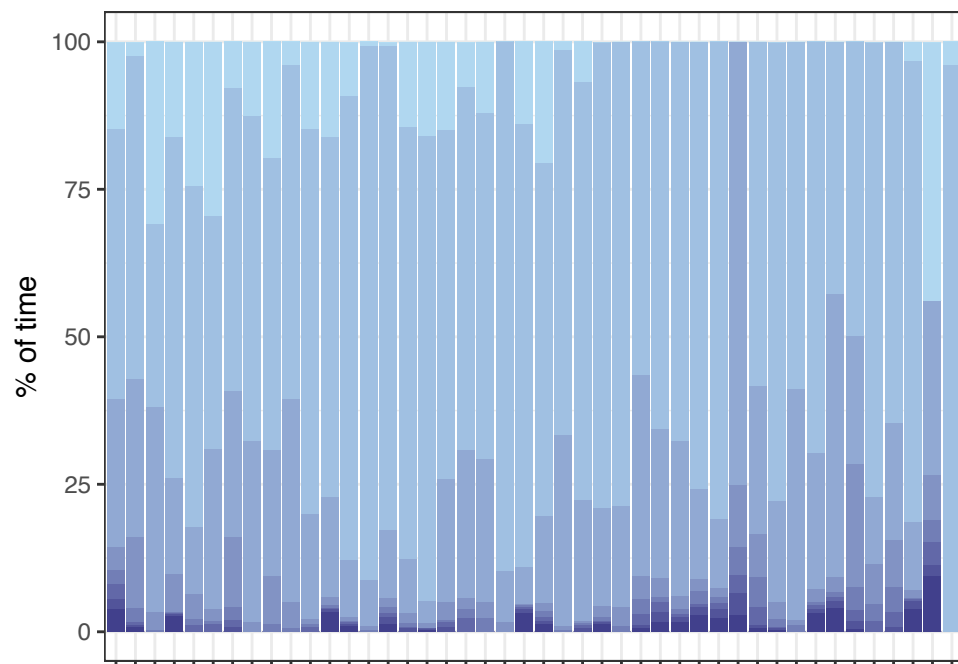

P - 821 Night

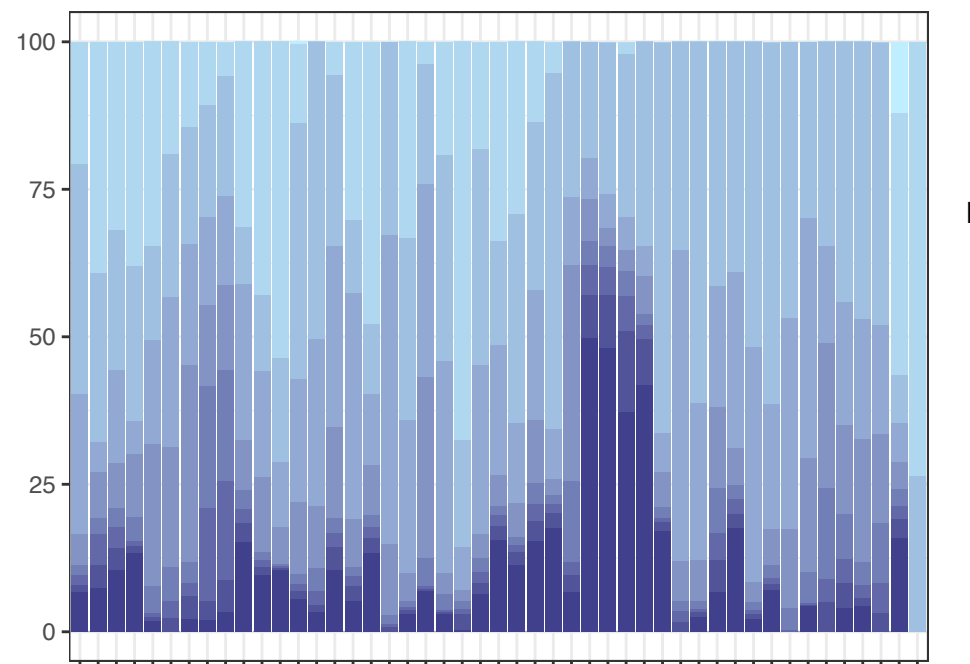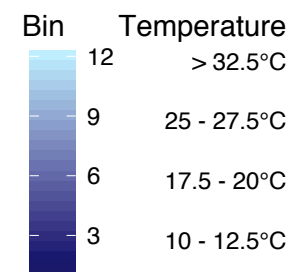

Supplement: Supplemental Information 9 [file peerj-06-5231-s009.pdf]

P - 955

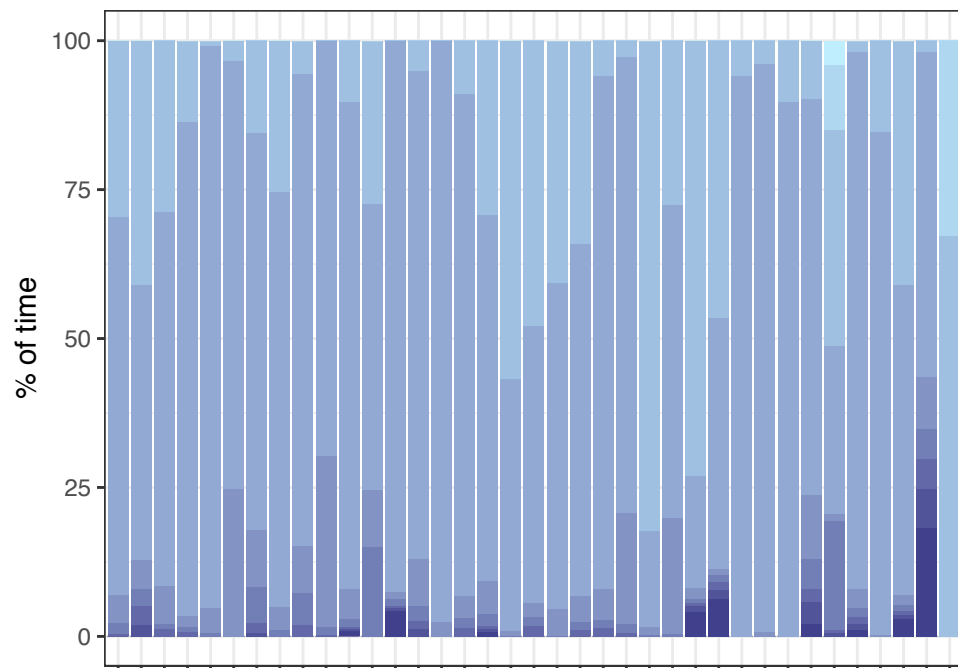

P - 955

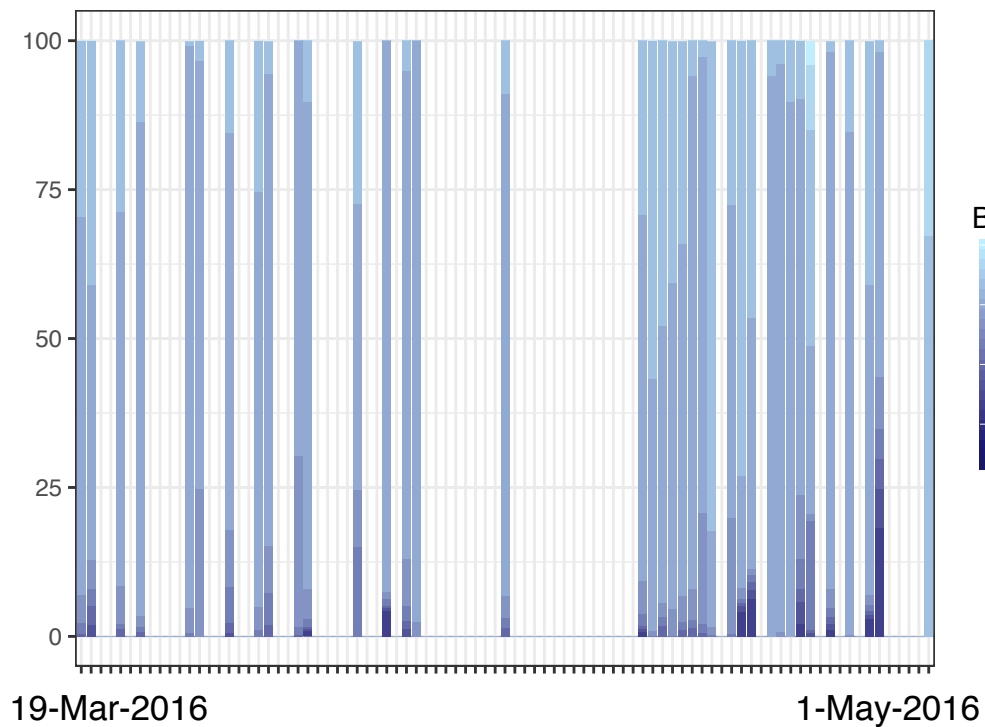

P - 955 Day

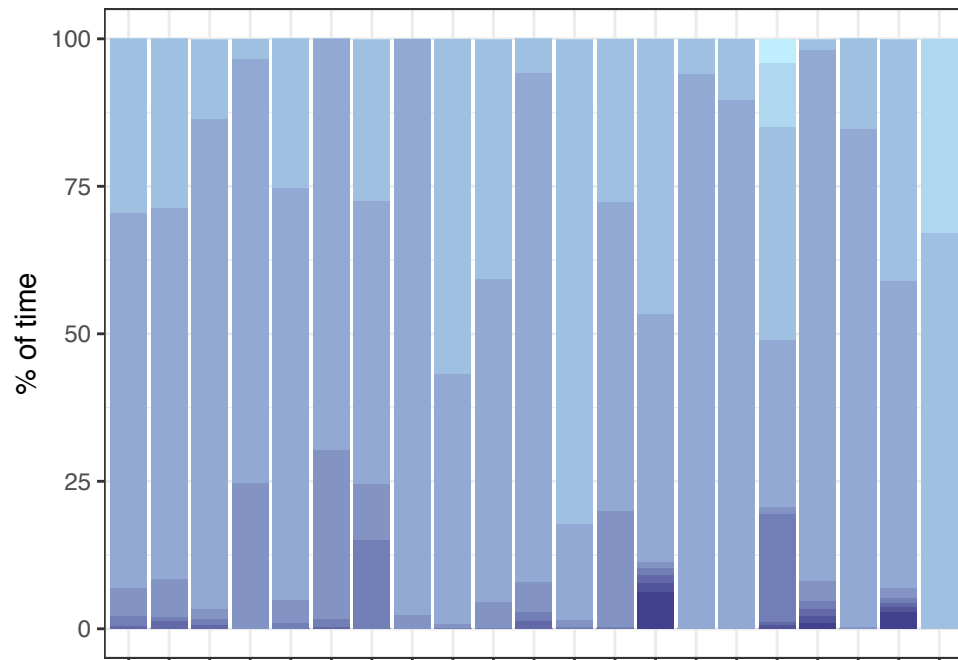

P - 955 Night

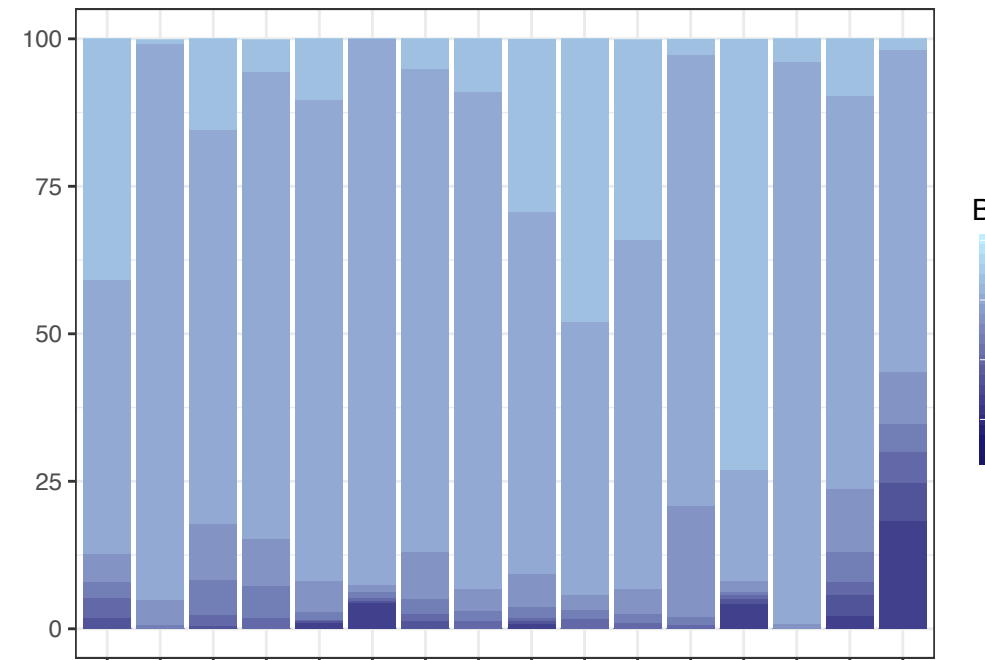

Supplement: Supplemental Information 10 [file peerj-06-5231-s010.pdf]

P - 813

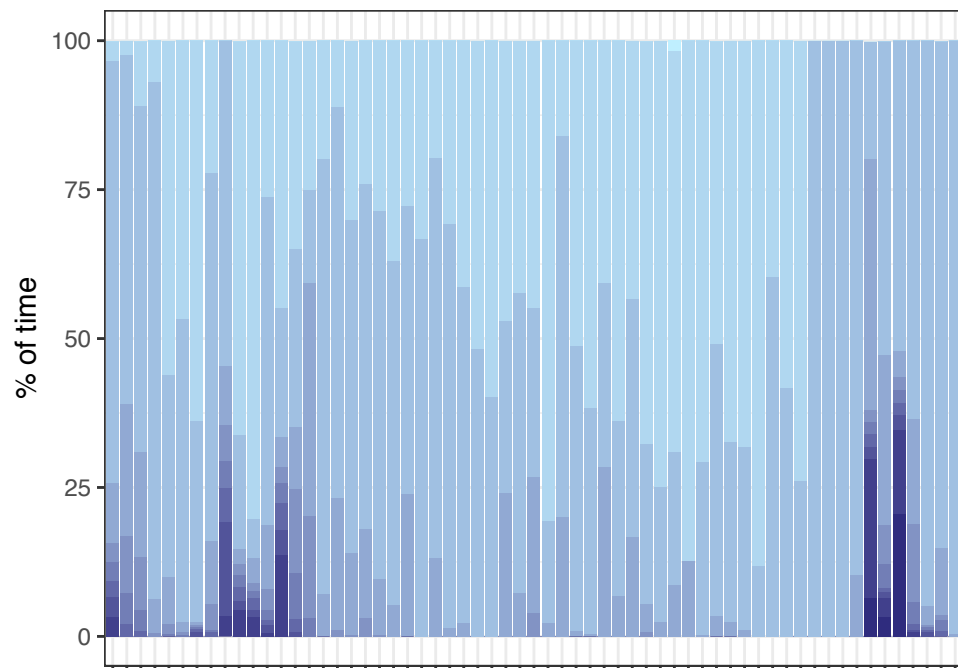

P - 813

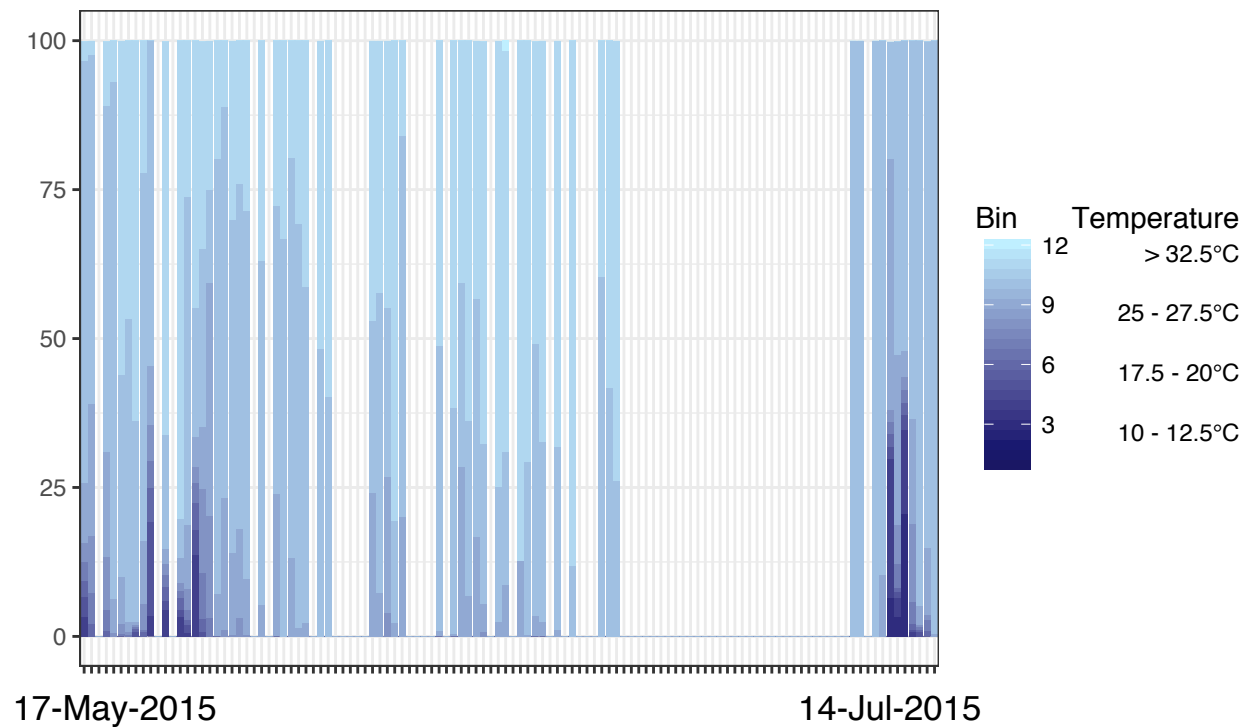

P - 813 Day

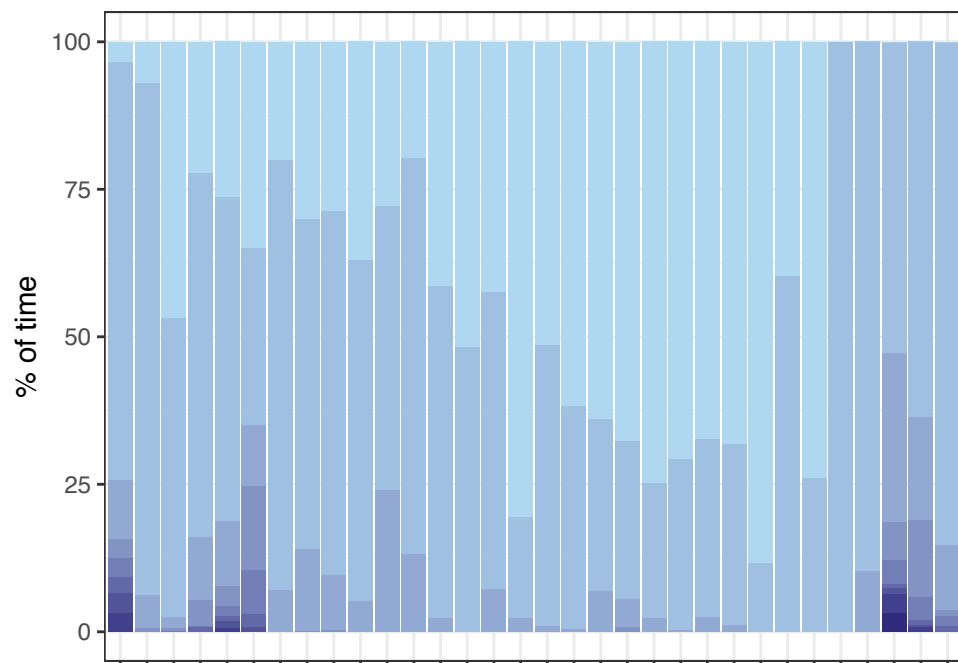

P - 813 Night

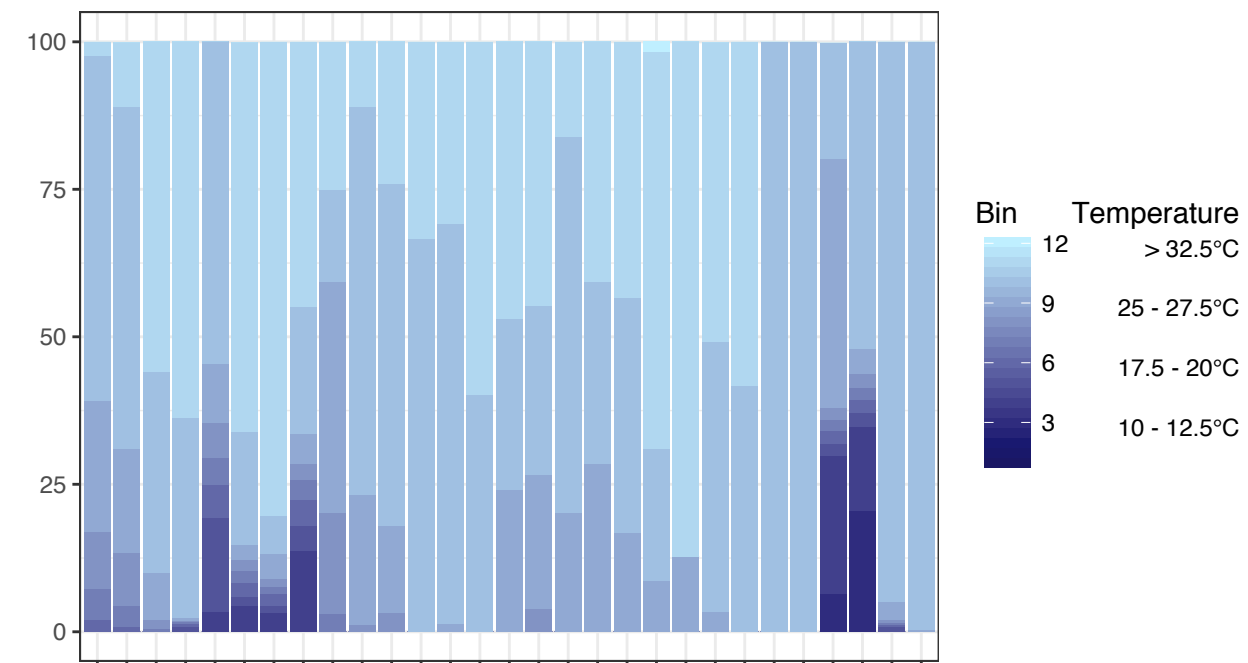

Supplement: Supplemental Information 11 [file peerj-06-5231-s011.pdf]

P – 905

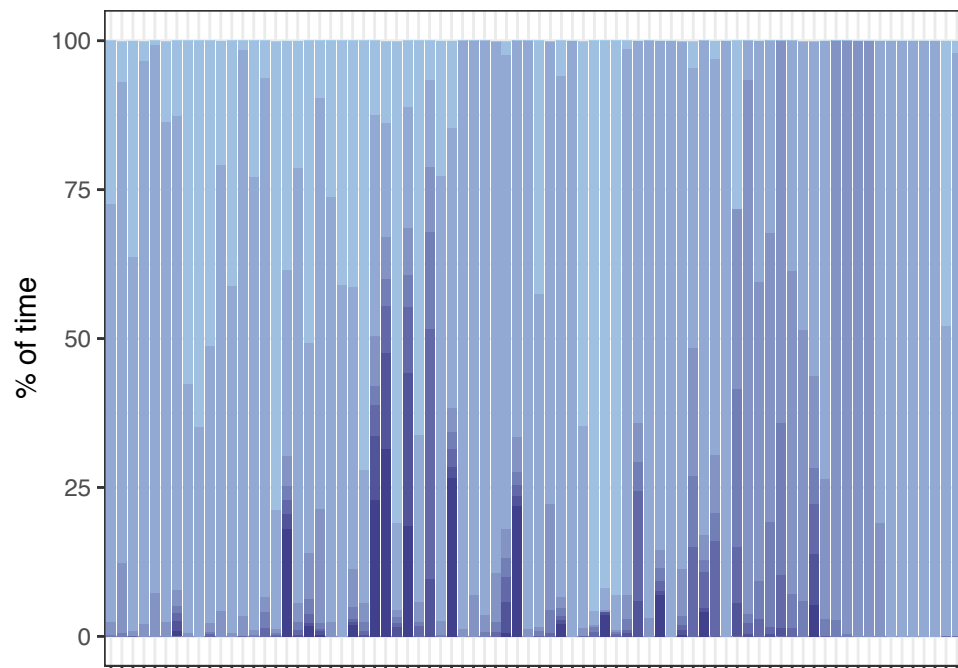

P – 905

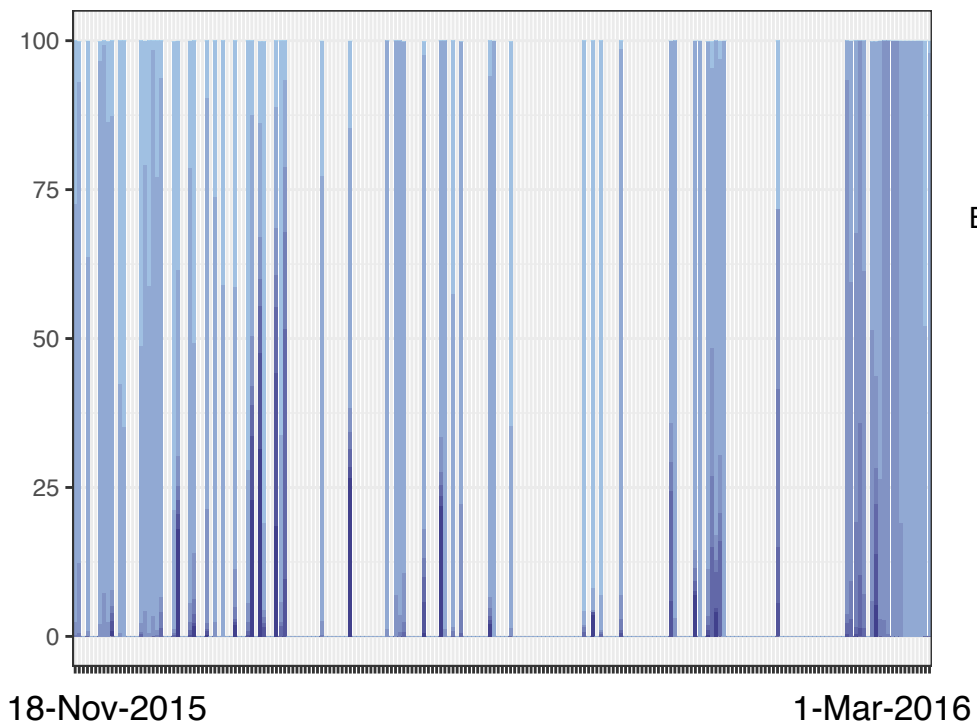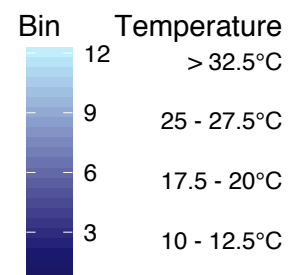

P – 905 Day

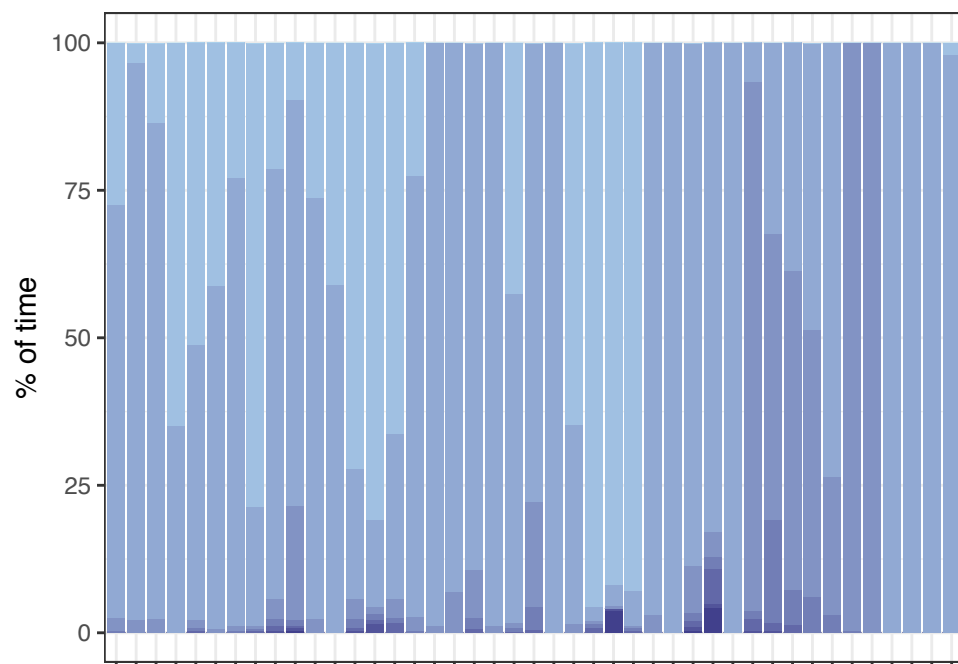

P – 905 Night

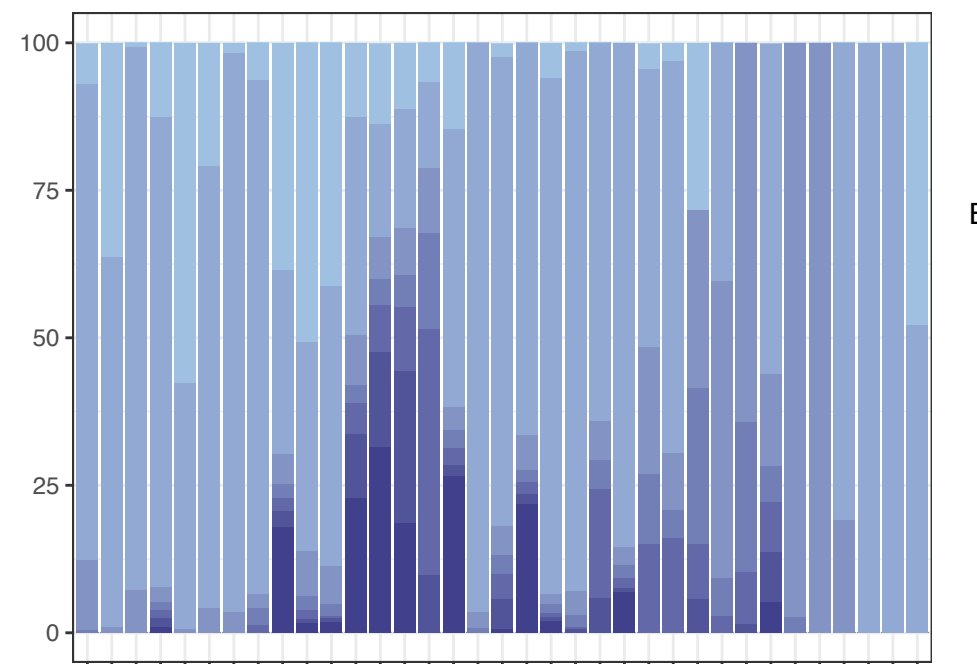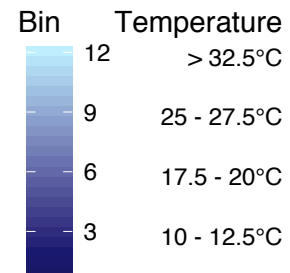

Supplement: Supplemental Information 12 [file peerj-06-5231-s012.pdf]

P - 971

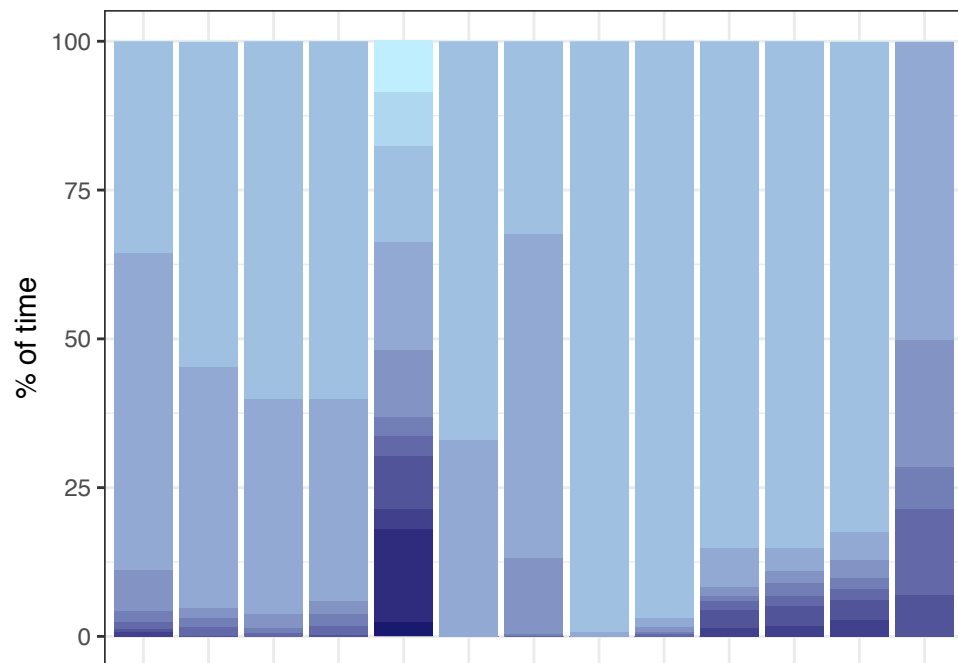

P - 971

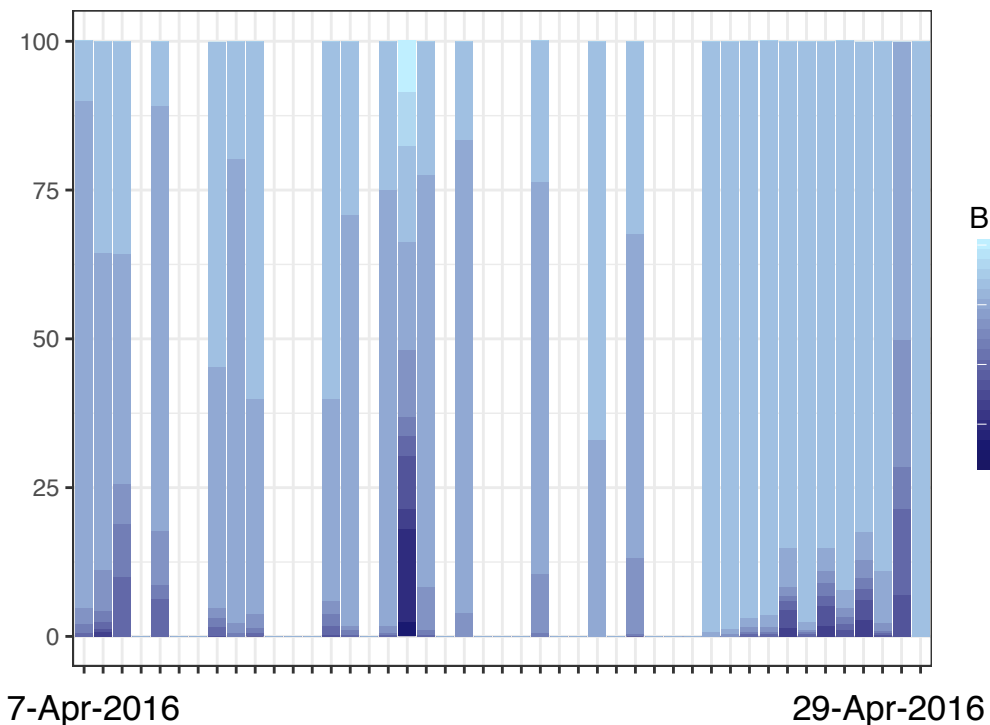

P - 971 Day

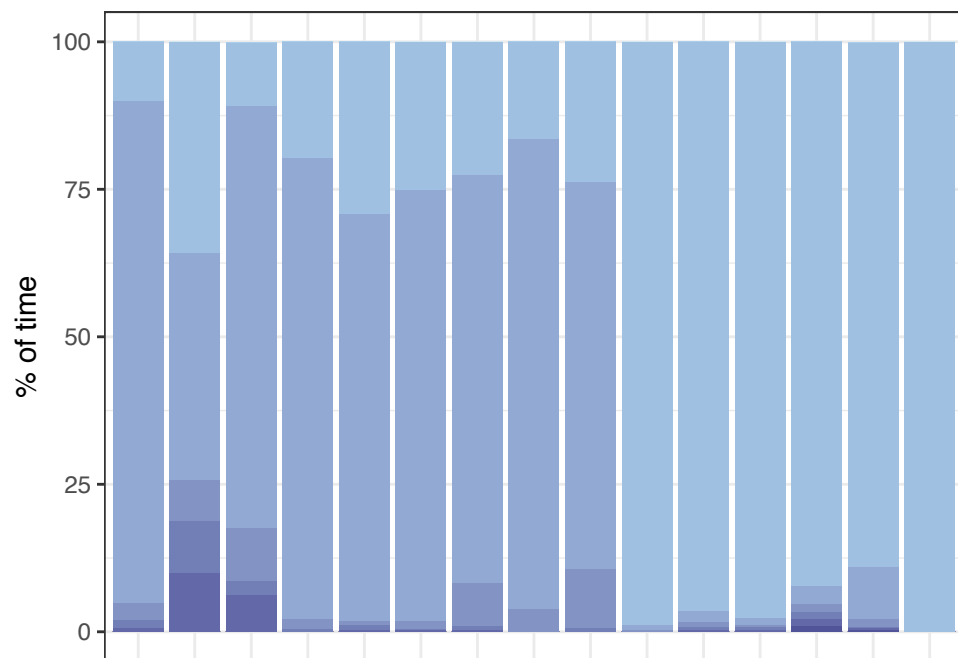

P - 971 Night

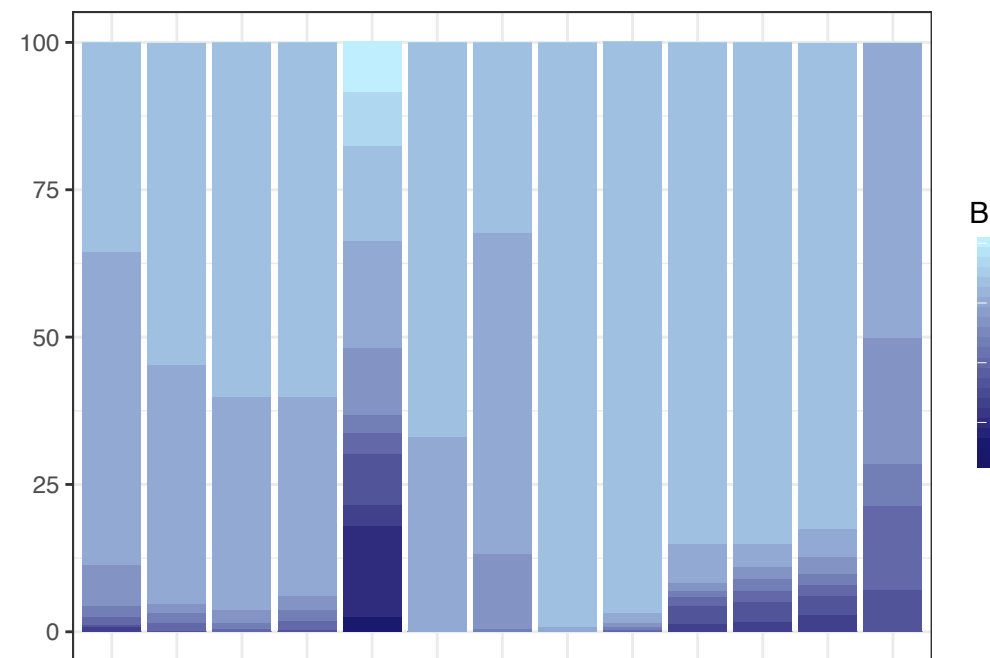

Supplement: Supplemental Information 13 [file peerj-06-5231-s013.pdf]

P - 970

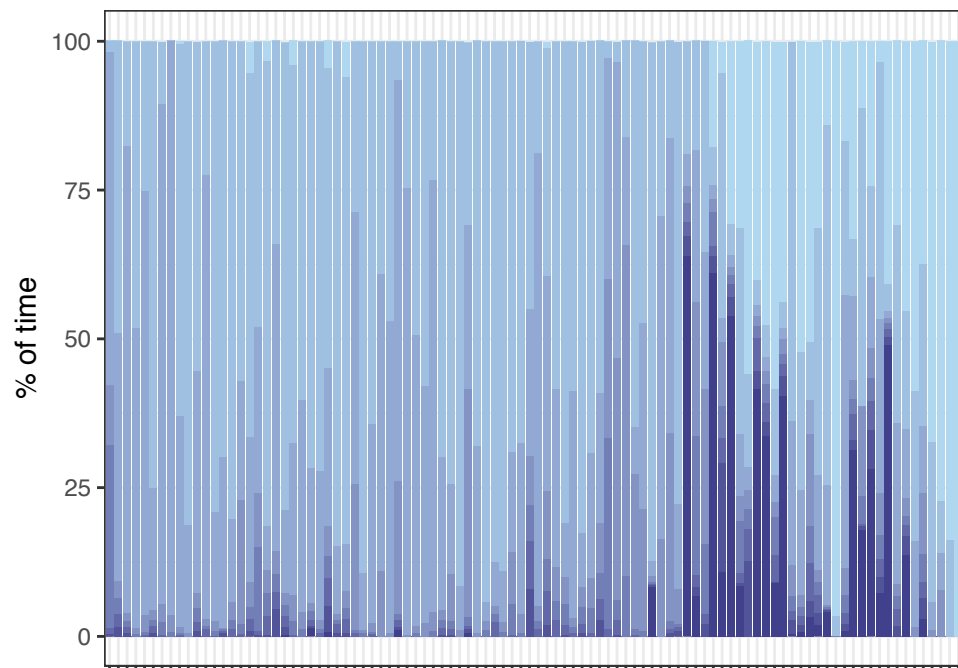

P - 970

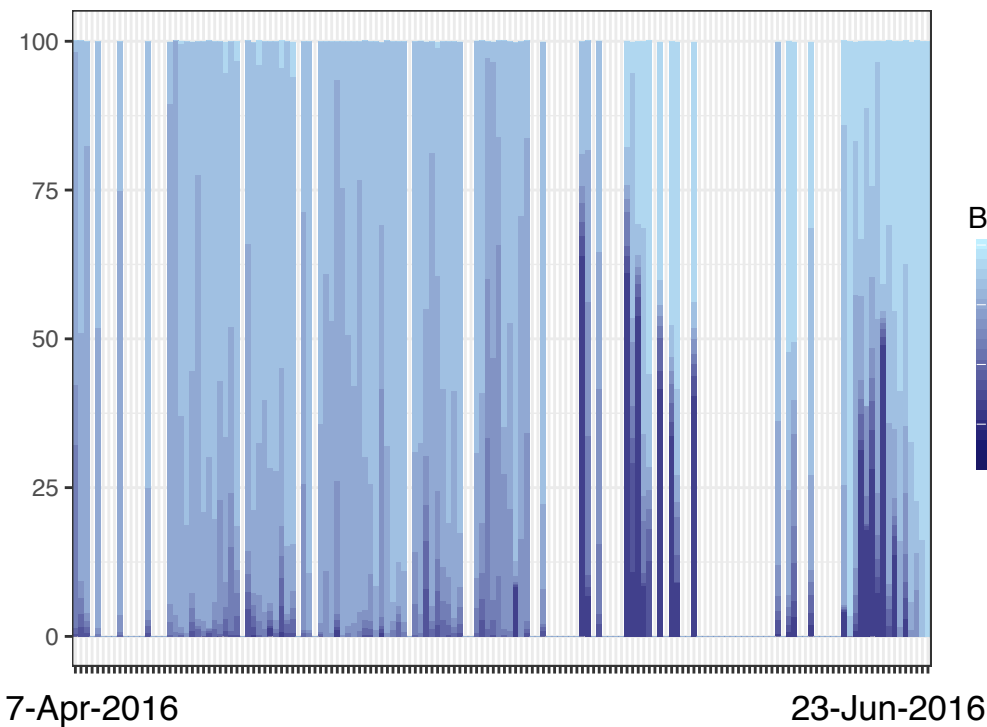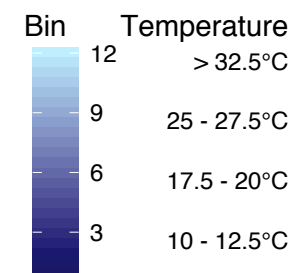

P - 970 Day

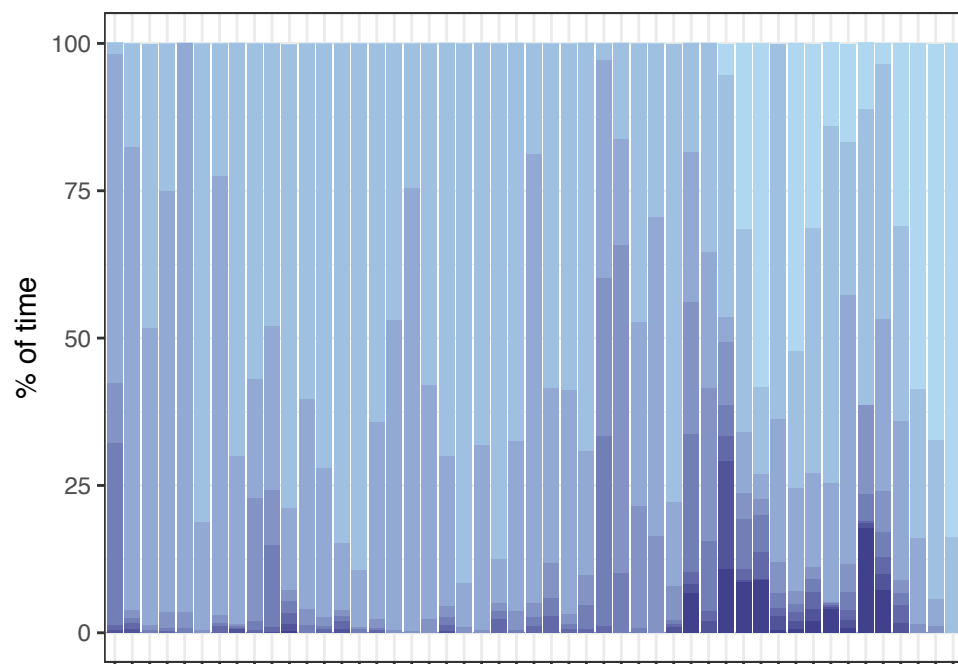

P - 970 Night

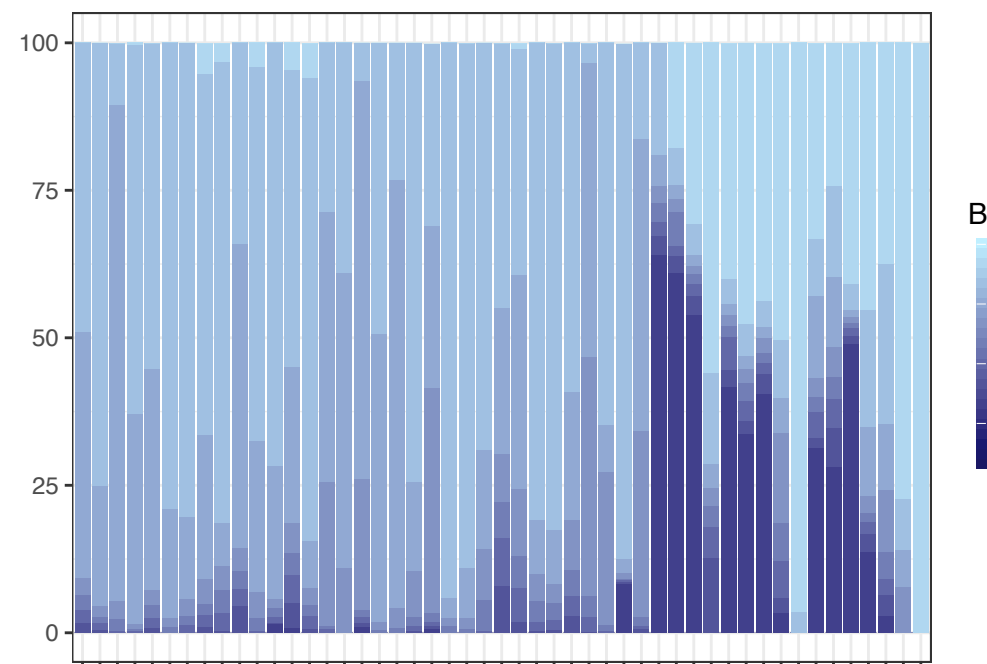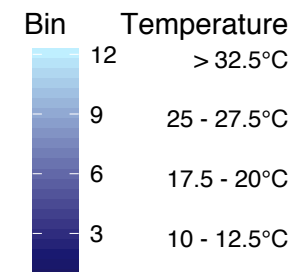

Supplement: Supplemental Information 14 [file peerj-06-5231-s014.pdf]

P - 491

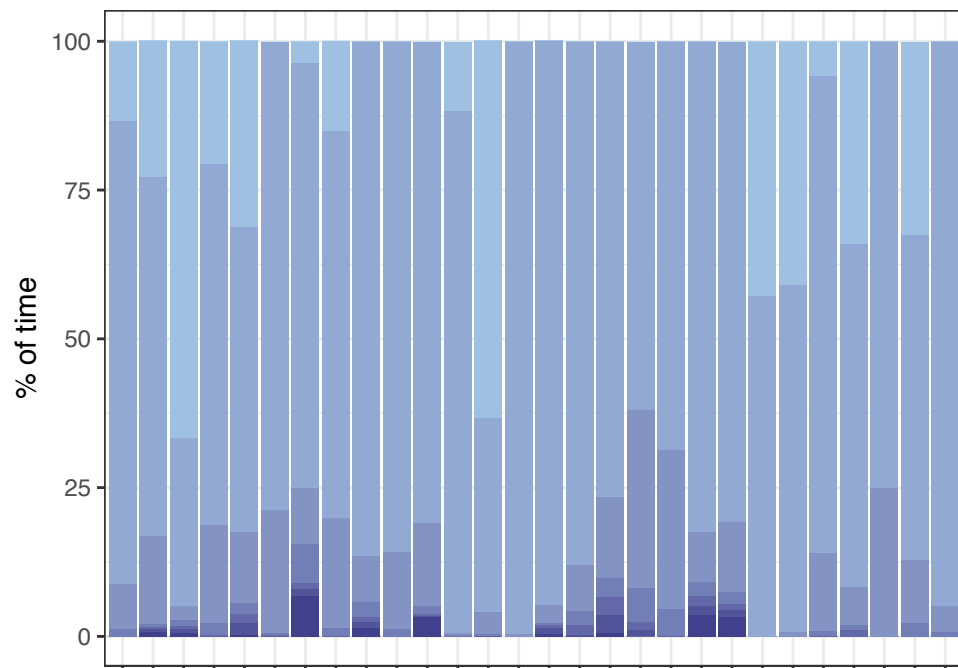

P - 491

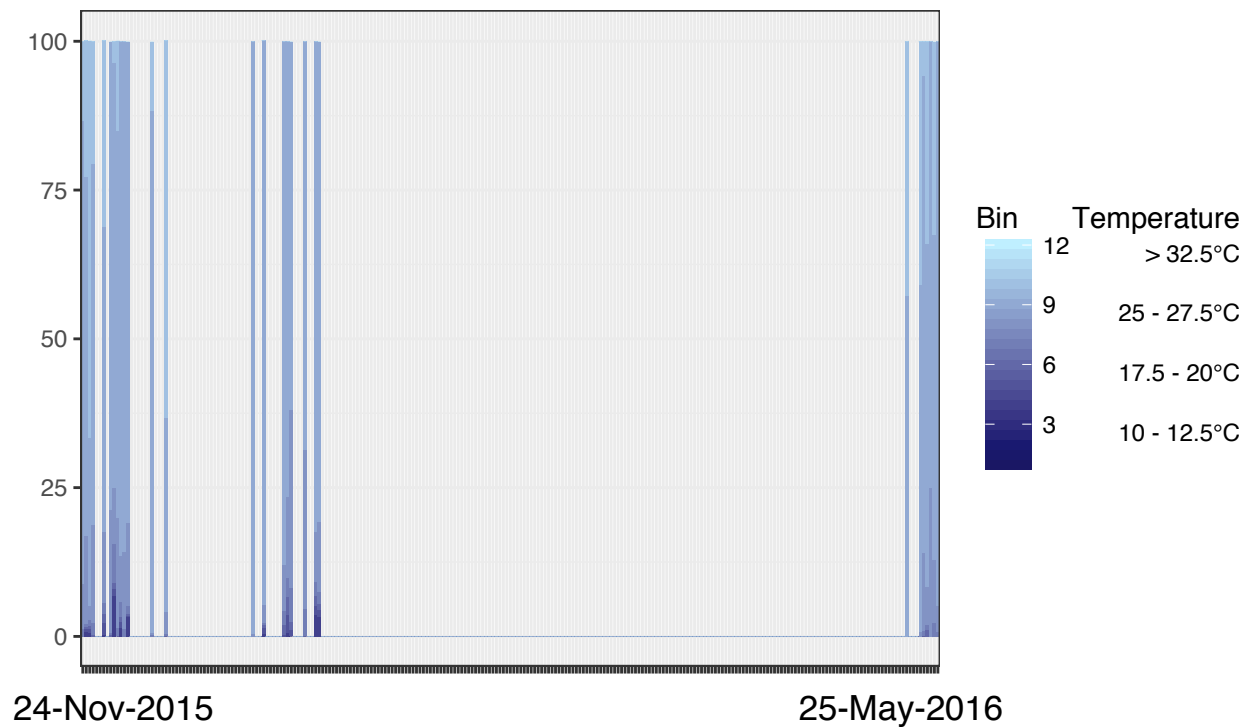

P - 491 Day

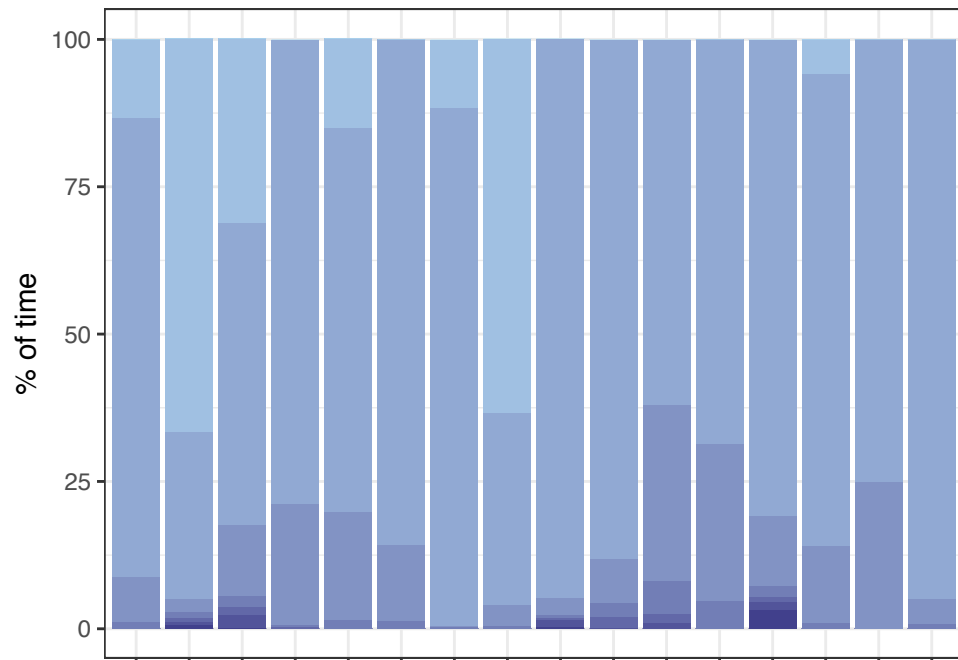

P - 491 Night

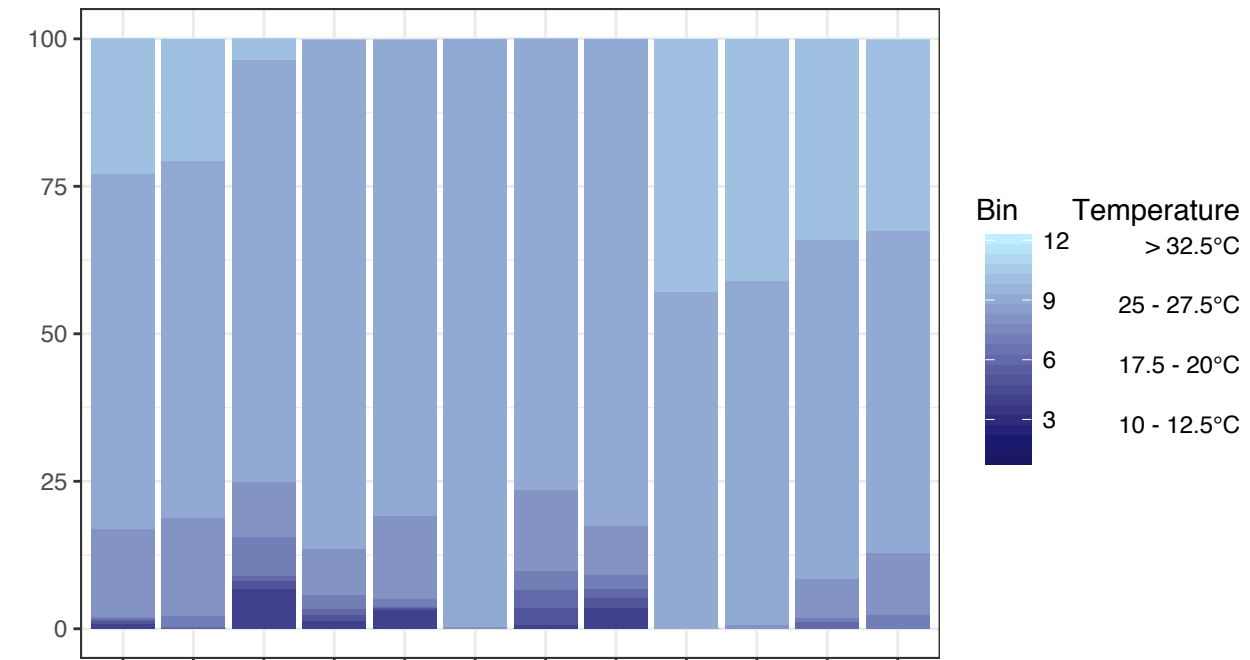

Supplement: Supplemental Information 15 [file peerj-06-5231-s015.pdf]

P - 926

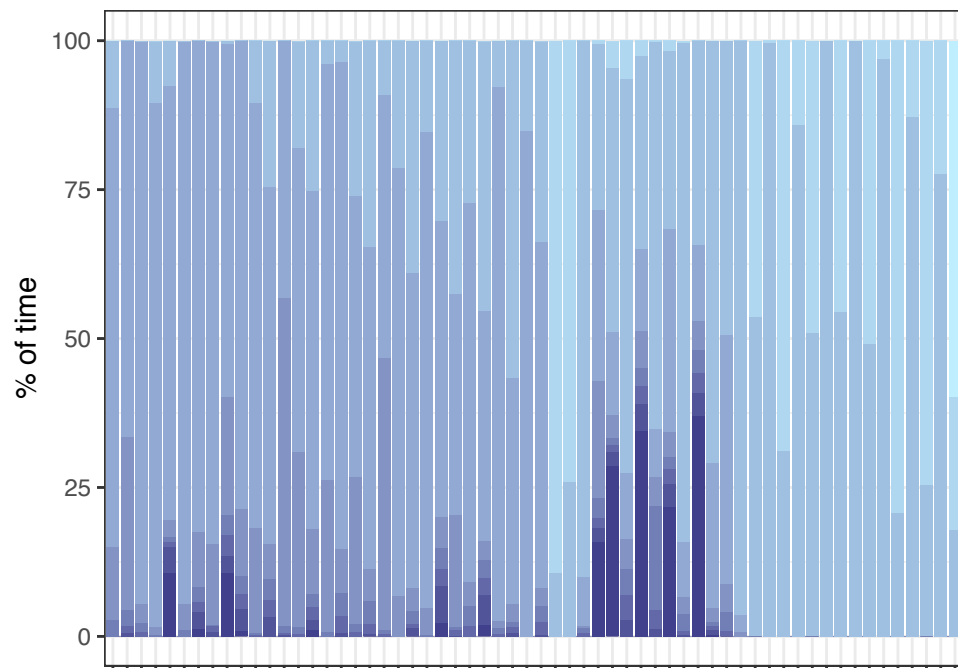

P - 926

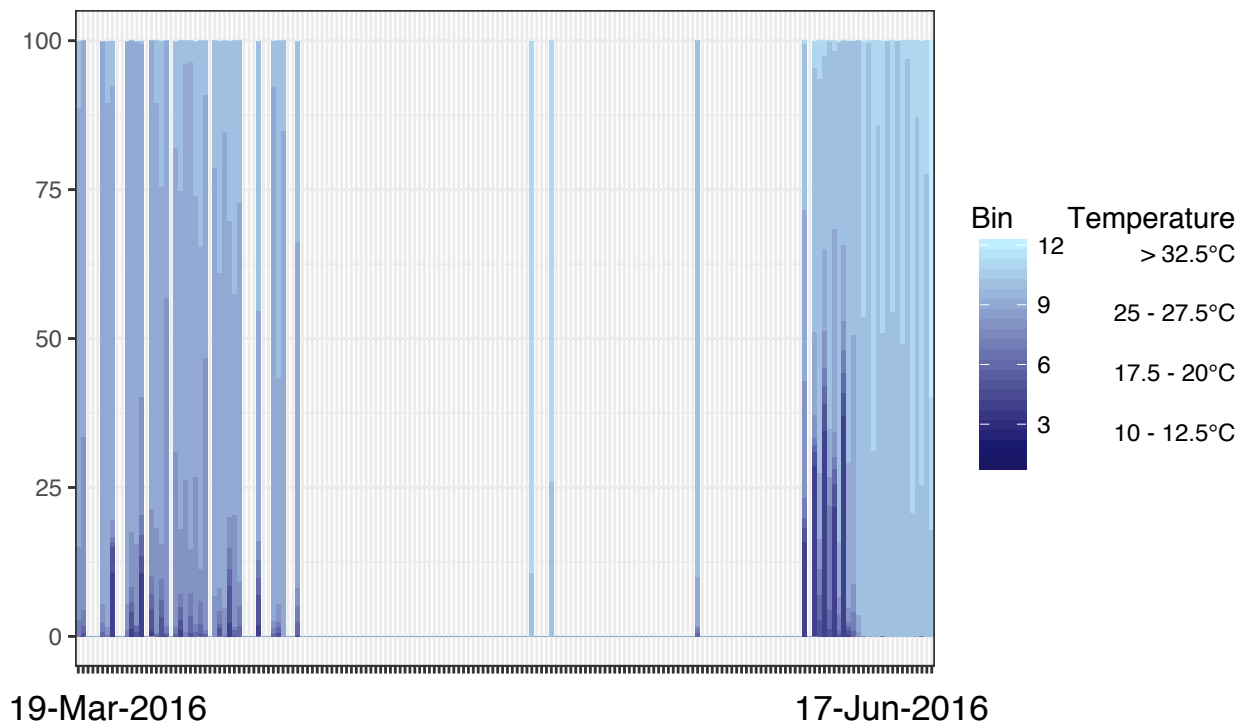

P - 926 Day

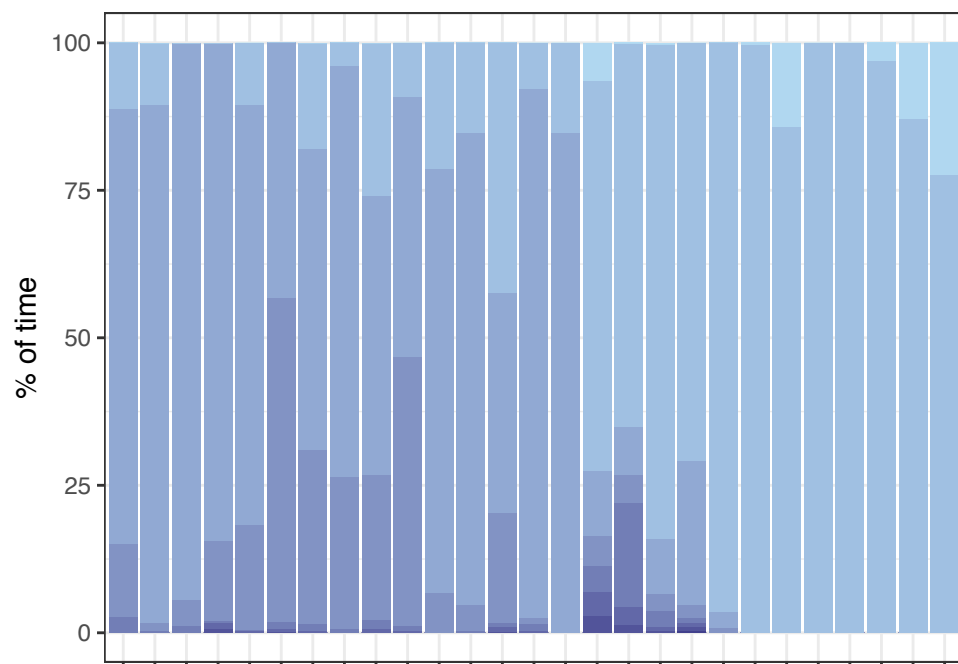

P - 926 Night

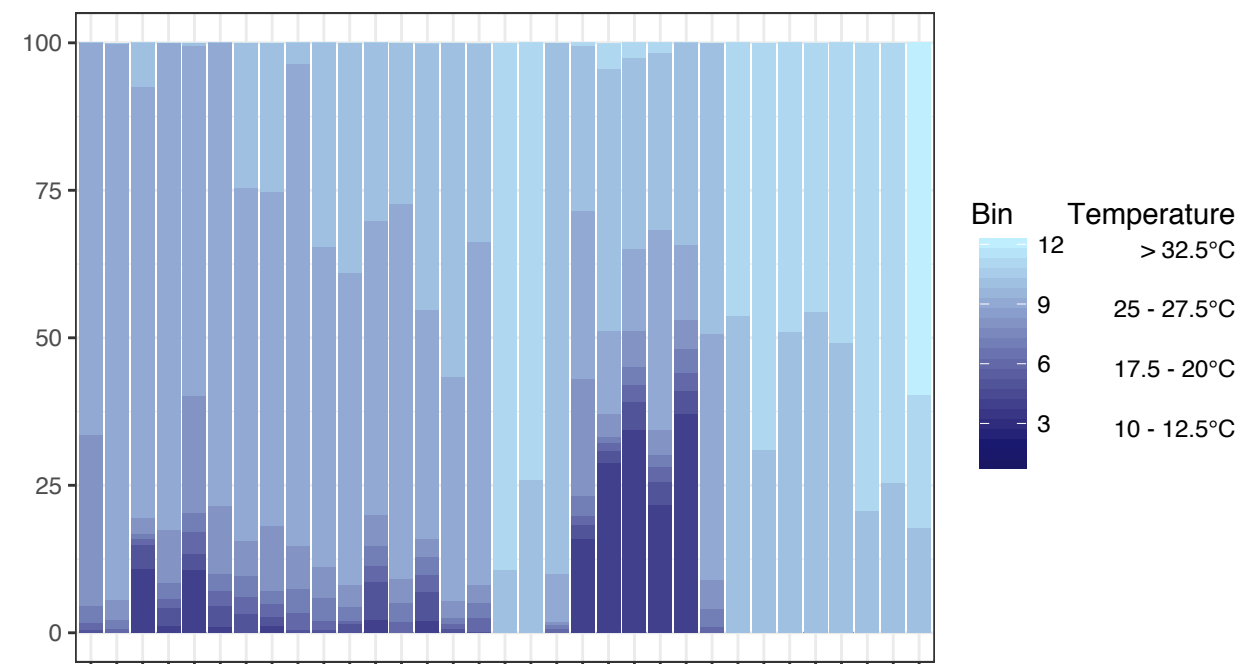

Supplement: Supplemental Information 16 [file peerj-06-5231-s016.pdf]
